# *In planta* study of photosynthesis and photorespiration using NADPH and NADH/NAD^+^ fluorescent protein sensors

**DOI:** 10.1038/s41467-020-17056-0

**Published:** 2020-06-26

**Authors:** Shey-Li Lim, Chia Pao Voon, Xiaoqian Guan, Yi Yang, Per Gardeström, Boon Leong Lim

**Affiliations:** 1 School of Biological Sciences, University of Hong Kong, Pokfulam, Hong Kong China; 2 Synthetic Biology and Biotechnology Laboratory, State Key Laboratory of Bioreactor Engineering, Shanghai Collaborative Innovation Center for Biomanufacturing Technology, East China University of Science and Technology, Shanghai, China; 3 Umeå Plant Science Centre, Department of Plant Physiology, Umeå University, SE-901 87 Umeå, Sweden; 4 State Key Laboratory of Agrobiotechnology, The Chinese University of Hong Kong, Hong Kong, China; 5 HKU Shenzhen Institute of Research and Innovation, Shenzhen, China

**Keywords:** C3 photosynthesis, Chloroplasts

## Abstract

The challenge of monitoring *in planta* dynamic changes of NADP(H) and NAD(H) redox states at the subcellular level is considered a major obstacle in plant bioenergetics studies. Here, we introduced two circularly permuted yellow fluorescent protein sensors, iNAP and SoNar, into *Arabidopsis thaliana* to monitor the dynamic changes in NADPH and the NADH/NAD^+^ ratio. In the light, photosynthesis and photorespiration are linked to the redox states of NAD(P)H and NAD(P) pools in several subcellular compartments connected by the malate-OAA shuttles. We show that the photosynthetic increases in stromal NADPH and NADH/NAD^+^ ratio, but not ATP, disappear when glycine decarboxylation is inhibited. These observations highlight the complex interplay between chloroplasts and mitochondria during photosynthesis and support the suggestions that, under normal conditions, photorespiration supplies a large amount of NADH to mitochondria, exceeding its NADH-dissipating capacity, and the surplus NADH is exported from the mitochondria to the cytosol through the malate-OAA shuttle.

## Abstract (web summary)

NADP(H) and NAD(H) are crucial energy molecules in plant metabolism. Here, via the use of circularly permutated fluorescent protein sensors, the authors demonstrate dynamic changes in NADPH and the NADH/NAD^+^ ratio during photosynthesis and photorespiration at the subcellular level *in planta*.

## Introduction

Adenosine triphosphate (ATP), nicotinamide adenine dinucleotide phosphate (NADPH), and nicotinamide adenine dinucleotide (NADH) are crucial energy molecules in living systems. Numerous studies have measured these metabolites in vitro, using bioluminescence, HPLC, mass spectrometry, enzymatic, and radioactive methods. However, in vitro methods require the extraction of ATP, NADPH, and NADH from tissues before measurements can be carried out^1^ and therefore cannot determine the instant levels of these metabolites in different subcellular compartments of different cells in a tissue.

Recently, our group has employed a novel MgATP^2−^-specific Förster resonance energy transfer (FRET)-based sensor to study the in vivo ATP levels of the plastids and cytosol of different tissues in *Arabidopsis thaliana* (Arabidopsis)^2^. A change in the FRET signal reflects a change in MgATP^2−^ concentration. By using this ATP sensor and a pH sensor, we have shown that the stromal ATP concentration is significantly lower than the cytosolic ATP concentration; that the import of ATP into mature chloroplasts is impeded by the downregulation of nucleotide transporters; that cytosolic ATP does not enter mature chloroplasts and is, therefore, unlikely to support the Calvin–Benson–Bassham (CBB) cycle; that, instead of ATP import, in Arabidopsis it is the export of reducing equivalents from chloroplasts that balances the NADPH/ATP supply from the linear electron flow and the NADPH/ATP demand of the CBB cycle^2^. Here, we employed two circularly permuted yellow fluorescent protein-based protein sensors, iNAP^3^ and SoNar^4^, to measure the *in planta* dynamic changes of NADPH and the NADH/NAD^+^ ratio induced by illumination in several subcellular compartments of Arabidopsis plants. These sensors were engineered from the bacterial Rex repressor capable of sensing pyridine dinucleotides redox states, and have many desirable properties such as intense fluorescence, rapid response, high specificity, and large dynamic range. Similar to the FRET based ATP sensor, the measurements of these sensors are also ratiometric and are less prone to the varied sensor expression levels in different tissues and compartments. We used stably transformed lines expressing these sensors to investigate photosynthesis and photorespiration.

Ribulose-1,5-bisphosphate carboxylase/oxygenase (Rubisco) carries out the initial step in the fixation of CO_2_ during photosynthesis. Rubisco is a slow enzyme, with a *k*_cat_ of 1–10 s^−1^ ^5^. Although it has a selectivity for CO_2_ over O_2_ of almost a factor of 100, due to the much higher concentration of O_2_ (21%) than CO_2_ (0.4%) in the atmosphere, it is estimated that for every three CO_2_ molecules fixed in C3 plants, about one O_2_ molecule is mistakenly utilized by Rubisco to generate one 3-phosphoglycerate (3-PG) and one 2-phosphoglycolate (2-PG) molecule^6,7^. The recycling of the photorespiration product 2-PG back to 3-PG involves eight enzymatic steps in chloroplasts, mitochondria, and peroxisomes. Hence, photorespiration has been regarded as a wasteful process due to the additional consumption of stromal ATP and liberation of CO_2_ in mitochondria in the cycle^8^.

During photorespiration, one NADH, one CO_2_, and one NH_3_ molecule are generated in the mitochondria through the conversion of two glycine molecules to one serine cooperatively by glycine decarboxylase (GDC) and serine hydroxymethyl transferase (SHMT). A certain amount of serine is exported to the cytosol for other uses^9^, whereas most is imported into peroxisomes and converted into hydroxypyruvate, which is then reduced to glycerate by hydroxypyruvate reductase (HPR), at the expense of NADH molecules. By converting the malate imported into the organelle, peroxisomal NAD-dependent malate dehydrogenase (NAD-MDH) provides the NADH required for hydroxypyruvate reduction^10^.

During photosynthesis, the linear electron flow (LEF) generates ATP and NADPH at a ratio of 1.28^11^, whereas the CBB cycle requires ATP and NADPH at a 1.5 ratio, resulting in a deficiency of ATP^12^ or an excess of NADPH^13^. Although the cyclic electron flow can contribute extra ATP, mature mesophyll chloroplasts are unable to import cytosolic ATP^2^. Hence, chloroplasts must recycle excess NADPH into NADP^+^ to serve as the electron acceptor during photosynthesis to balance the ratio of stromal ATP to NADPH.

In chloroplasts, NADP^+^ is recycled from NADPH via three major routes: the CBB pathway and 3-phosphoglyceric acid/triose phosphate (PGA/TP) exchange coupled with it, fatty acid synthesis, and the malate-oxaloacetate (malate-OAA) shuttle. Excess NADPH generated from the LEF can be exported to the cytosol through the shuttle in the form of malate via the action of NADP-dependent malate dehydrogenase (NADP-MDH)^14,15^, which converts OAA into malate and regenerates NADP^+^^16^. Hence, during daytime, malate builds up in leaf cells, where most of it is stored in the vacuole, whereas the cytosolic malate concentration is maintained at a relatively constant, low level^17^. Some cytosolic malate is imported into the peroxisomes, where it is used by peroxisomal NAD-MDH to recycle OAA and regenerate the NADH required for hydropyruvate reduction^10^. In mitochondria, both the malate-OAA shuttle^12,16,18–22^ and photorespiration^23,24^ have been proposed to provide reducing equivalents to the mitochondrial electron transport chain (mETC) for ATP production. In a flux balance model of the mature leaves of C3 plants, both photorespiration and the malate-OAA shuttle are predicted to contribute to feeding NADH into the mETC^25^. Experimental data obtained from barley leaf protoplasts^23,24,26^ and isolated mitochondria^27^ suggest that photorespiration is the major source of reducing equivalents to the mETC. However, this has not been examined in a whole plant level, and the direction of the flow of reducing equivalents between different subcellular compartments during photosynthesis has not yet been fully resolved^28^.

Here, by employing a NADPH and a NADH/NAD^+^ sensors, we examined *in planta* dynamic changes in the NADPH pools and NADH/NAD^+^ ratio in the stroma and cytosol upon illumination. We suggest that, at the light intensities we used, photorespiration supplies a large amount of reducing equivalents to mitochondria during photosynthesis, which exceeds the NADH-dissipating capacity of the mETC. Consequently, the surplus NADH must be exported from the mitochondria to the cytosol through the mitochondrial malate-OAA shuttle.

## Results

### Establishing iNAP and SoNar sensors in Arabidopsis

We generated multiple transgenic Arabidopsis lines in the wild-type (WT, Col-0) background expressing a high-affinity NADPH sensor (iNAP1), a low-affinity NADPH sensor (iNAP4), and an NADH/NAD^+^ sensor (SoNar) in the cytosol (Supplementary Fig. 1). These cytosolic sensors were occasionally seen in the nucleus in some tissues (e.g. guard cells). We also fused the iNAP1, iNAP4, and SoNar sensors with organelle-specific targeting peptides to direct their expression specifically to the plastid stroma and peroxisome. In addition, we generated Arabidopsis lines expressing iNAPc (Supplementary Fig. 1f–h), in which the ligand binding to pyridine nucleotides is fully abolished in each of these compartments, to serve as a pH control^3^. At least two independent lines expressing each sensor in each compartment were selected for analysis. No abnormal phenotype was observed for these lines, and the plant size did not show significant differences among lines (Supplementary Fig. 1i, j). We imaged the seedlings using a confocal microscope configured to detect chlorophyll autofluorescence (excitation at 488 nm and emission at 629–700 nm). The presence of iNAP and SoNar fluorescence in the peripheral cytoplasm indicated that these sensors were expressed in the cytosol (Supplementary Fig. 2a), whereas the fluorescence signals of TKTP-iNAP and TKTP-SoNar (fusions of the plastid-specific chloroplast transketolase transit peptide, TKTP, from *Nicotiana tabacum*) co-localized with chlorophyll autofluorescence, corroborating the chloroplast expression of both sensors (Supplementary Fig. 2b). We also compared the distribution of iNAP4-SRL and iNAPc-SRL (fusions of iNAP4 and iNAPc with the peroxisome marker peroxisomal targeting signal type 1, SRL) with that of mRFP-SRL (a fusion of red fluorescent protein with SRL) using transient protoplast transformation. Both iNAP4-SRL and iNAPc-SRL co-localized with mRFP-SRL in a typical peroxisome pattern visualized in spherical spots, suggesting that both sensors were targeted specifically to peroxisomes (Supplementary Fig. 2c). However, no Arabidopsis lines expressing SoNar-SRL were obtained after repeated transformation attempts. We also failed to introduce these sensors into mitochondria of WT and *suppressor of gene silencing 3-13* (*sgs3-13*)^29^ lines using the presequence that successfully targeted GFP and the pH sensor cpYFP to mitochondrial matrix (Supplementary Fig. 2d)^30,31^.

### NADPH levels are lower in cytosol than in plastid stroma and peroxisome

**Fig. 1:**
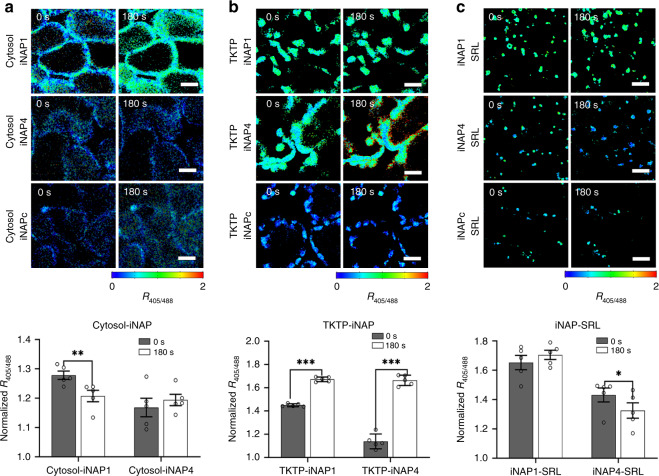
Light responses of iNAP sensors in different compartments of 10-day-old cotyledon mesophyll. **a** Seedlings expressing cytosolic iNAP1, iNAP4, and iNAPc. **b** Seedlings expressing stromal iNAP1, iNAP4, and iNAPc, and **c** seedlings expressing peroxisomal iNAP1, iNAP4, and iNAPc were continuously illuminated for 180 s at 296 μmol m^−2^ s^−1^ (*n* = 5; error bars ± SEM). Emissions at 520 nm after sequential excitation at 405 nm and 488 nm were recorded, and the ratios of the two emissions (*R*_405/408_) are presented in pseudocolor image, where high ratios (red) correspond to high NADPH levels. Scale bars, 20 μm. Asterisks indicate statistical significance as determined using paired *t*-test: *P < 0.05, **P < 0.01, ***P < 0.001.

To identify the iNAP sensors with the most suitable affinity for each compartment, we introduced iNAP sensors with high (iNAP1) and low (iNAP4) NADPH affinities into the cytosol, plastid stroma, and peroxisome. Since the iNAP1 and iNAP4 sensors are highly specific to NADPH^3^, and NADPH is produced during photosynthesis, the response of these sensors can be studied by illuminating the plants. Illumination at 296 µmol m^−2^ s^−1^ did not significantly affect the cytosolic iNAP4 ratio (Fig. 1a), but it decreased the cytosolic iNAP1 ratio significantly (*P* < 0.01), suggesting that the cytosolic NADPH pool was diminished upon exposure to light. Illumination caused significant (*P* < 0.001) ratiometric shifts for both the TKTP-iNAP4 and TKTP-iNAP1 sensors (Fig. 1b). However, TKTP-iNAP4 exhibited a greater ratio increment, suggesting that the iNAP1 signal was saturated upon illumination; we therefore used TKTP-iNAP4 throughout the remainder of our study.

**Fig. 2:**
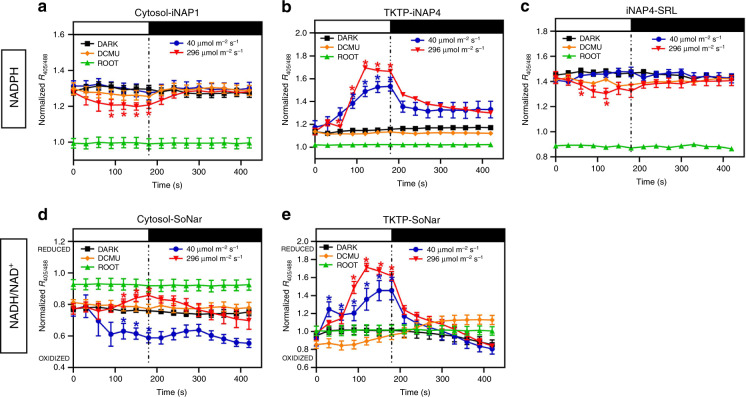
Light responses of iNAP and SoNar sensors in untreated cotyledon with 40 µmol m^−2^ s^−1^ or 296 µmol m^−2^ s^−1^ illumination or cotyledon pre-treated with DCMU or root at 296 µmol m^−2^ s^−1^ illumination. The normalized *R*_405/488_ shifts of **a** cytosolic iNAP1, **b** stromal iNAP4, **c** peroxisomal iNAP4, **d** cytosolic SoNar, and **e** stromal SoNar in mesophyll of 10-day-old cotyledon in response to illumination with an interval of 30 s are shown. Illumination up to 180 s is represented by white bars, and the period after light was turned off is indicated by black bars (*n* = 5; error bars ± SEM). All results shown were normalized with cytosol-iNAPc, TKTP-iNAPc, and iNAPc-SRL, respectively. Asterisks indicate that the differences (*P* < 0.05) between results from the dark condition and 40 µmol m^−2^ s^−1^ or 296 µmol m^−2^ s^−1^ illumination are statistically significant as determined by a paired *t*-test. DCMU, 3-(3,4-dichlorophenyl)-1,1-dimethylurea.

In peroxisomes, illumination caused a significant decrease (*P* < 0.05) in the ratio of the low-affinity iNAP4-SRL sensor lines but not the high-affinity iNAP1-SRL lines (Fig. 1c), indicating that the NADPH concentration in peroxisomes exceeded the maximum dynamic limit of the iNAP1 sensor. Similar responses were not observed in illuminated roots, suggesting that the responses observed in the mesophyll were due to photosynthesis (Fig. 2). Together, the differences in saturation response between iNAP1 and iNAP4 in these compartments suggest that the NADPH level in the cytosol is lower than those in plastid stroma and peroxisome in Arabidopsis. For further *in planta* analyses, we employed cytosolic iNAP1, TKTP-iNAP4, and iNAP4-SRL Arabidopsis lines.

To test the in vitro and semi in vivo characteristics of the sensor proteins, we adopted recombinant proteins and seedling root tips to monitor the specificity of NADPH and NADH/NAD^+^ binding affinity curves. The in vitro *K*_d_ values determined for iNAP1 and iNAP4 at 22 °C were 0.3 μM and 30 μM, respectively, which were much lower than the values 2.0 μM and 120 μM obtained at 37 °C^3^. The *K*_NADH/NAD+_ of SoNar at 22 °C was determined to be 0.04 (Supplementary Fig. 3), which was slightly higher than the value (0.025) obtained at 37 °C^32^. In semi in vivo conditions, the sensors displayed increasing ratios at a higher level or ratio of exogenous nucleotides (Supplementary Fig. 4), where the *K*_d_ values for iNAP1, iNAP4, and *K*_NADH/NAD+_ for SoNar were calculated as 2.5, 5.6, and 1.48 μM, respectively, which were different from the values obtained in vitro. However, the values determined by the semi in vivo protocol might be affected by possible artifacts. First, there are endogenous NADPH in the root cells and zero value could not be set. As a result, the *K*_d_ value for iNAP1 would be overestimated. Second, the ratios of many cells in the root tips were averaged for ratio calculation but the degree of penetration of exogenous NADPH into different cells varied. This could lead to an underestimation of the *K*_d_ value for iNAP4. Nonetheless, the semi in vivo experiments showed that the sensors did response in vivo.

### Ratiometric imaging of subcellular NADPH level and NADH/NAD^+^ ratio

Prior to analyses, we validated two independent lines expressing each sensor. We tested different confocal laser powers to ensure that the laser power at 405 nm was sufficient and the illumination was not depleted by chlorophyll absorption. Raw *R*_405/488_ ratios obtained from 3-day-old seedlings of two independent iNAP and SoNar (cytosol, TKTP, and SRL) lines with different confocal laser powers indicated that, within the same tissues, the ratios were practically identical in all of the independent lines, with no statistically significant differences (Supplementary Fig. 5). These lines stably expressed sensors with different fluorescence intensities and thus different sensor concentrations in the same compartment, but different lines emitted similar ratios under untreated conditions. We selected the sensor lines with higher fluorescence intensities to use in our subsequent *in planta* studies (Line 1 for TKTP-iNAP4, cytosolic SoNar, and TKTP-SoNar and Line 2 for cytosolic iNAP1 and iNAP4-SRL, respectively). Ratiometric analysis allows measurement of sensors independently of their expression levels, provided that there is a sufficient signal-to-noise ratio and little interference from any potential autofluorescence. The autofluorescence channel at the UV light spectra (405 nm excitation and 450 ± 17 nm emission) was also set to exclude any potential interference from plant lignin autofluorescence throughout the experiment.

To assess the validity of the collected emission spectra for seedlings expressing iNAP and SoNar, we scanned the emission range for iNAP and SoNar from 424 to 599 nm in 10-day-old seedlings with 405 nm and 488 nm excitation, respectively (Supplementary Fig. 6). As all iNAP and SoNar sensors showed a characteristic emission peak at 520 ± 20 nm, which was not seen in the WT, the fluorescence emission at 520 ± 20 nm was significantly lower in the WT control without sensors. Due to the capacity of chloroplasts to absorb blue wavelength light, we tested different laser powers to avoid possible interference from chloroplasts (Supplementary Fig. 5). Chlorophyll absorption indeed interferes with the ratio measurement, and therefore the ratios in chlorophyll-containing tissues (e.g., shoots) cannot be compared with those in tissues with less or no chlorophyll (e.g., root). Nonetheless, the dynamic changes in the sensor ratios in the same compartment under various treatments (e.g., light or inhibitors) can be followed. Moreover, the circularly permuted yellow fluorescent protein-based iNAP sensors are pH sensitive when it is excited at 488 nm but not at 405 nm^3,4^, and it is almost certain that plants have numerous cation exchangers that are localized in different membranes and that may regulate pH changes across the organelles in response to stimuli. Thus, pH effects need to be carefully normalized, which can be done using the pH control sensor (iNAPc) in parallel experiments. We therefore included an iNAPc control for each subcellular compartment in all measurements to normalize the effects derived from pH interference and chlorophyll absorption (Supplementary Fig. 7).

To determine the redox ratios potentially that could be achievable by the sensors, we exogenously applied the oxidizing agents menadione and hydrogen peroxide (H_2_O_2_) and the reducing agent dithiothreitol (DTT) by 5-min infiltration (Supplementary Fig. 8). Menadione and H_2_O_2_ are broadly used to oxidize plant cells, and both mediate ROS formation through the catalytic transfer of electrons from endogenous donors to molecular oxygen. To assess whether these agents might affect the sensors directly, we first carried out an in vitro experiment. Although H_2_O_2_ and menadione had no effect on the sensor ratios (Supplementary Fig. 8), DTT directly suppressed the ratios. Hence, only the *in planta* results for the oxidizing agents were shown. Menadione and H_2_O_2_ significantly reduced the ratios of both iNAP sensors in all three compartments, indicating that the NADPH levels in these three compartments in the absence of oxidizing treatment were higher than the lowest detection ranges of the iNAP sensors. Menadione and H_2_O_2_ treatments significantly lowered the SoNar ratio in plastid stroma but not in the cytosol (Supplementary Fig. 8), implying that the NADH/NAD^+^ ratio is very low in the cytosol in darkness. In fact, a highly oxidized NAD(H) pool was determined in the cytosol of pea protoplasts^33^ and the NADH/NAD^+^ ratio was lower in the cytosol than the chloroplasts of barley protoplasts^34^. The ratios stimulated by illumination at 296 µmol m^−2^ s^−1^ were used as controls (Supplementary Fig. 8). We also showed that l-ascorbic acid and reduced glutathione, which are abundant antioxidants in plant cells^35^, did not affect the ratios of the sensors in in vitro binding studies (Supplementary Fig. 9).

### Dynamic changes of NADPH and NADH/NAD^+^ under illumination

To examine the effect of light intensities, we compared the dynamic changes in subcellular pyridine nucleotides at two light intensities, 40 µmol m^−2^ s^−1^ and 296 µmol m^−2^ s^−1^. We observed significant increases in stromal NADPH and NADH/NAD^+^ and cytosolic NADH/NAD^+^, and decreases in cytosolic NADPH and peroxisomal NADPH, upon illumination at 296 µmol m^−2^ s^−1^ (Fig. 2). However, at 40 µmol m^−2^ s^−1^, the NADPH ratiometric shift occurred only in stroma but not in the cytosol or peroxisome. In addition, both the stromal NADH/NAD^+^ (Fig. 2e) and the cytosolic NADH/NAD^+^ were more oxidized (Fig. 2d) at the lower light intensity than at the higher light intensity, indicating that the redox status of the NADH/NAD^+^ pool in the cytosol is connected to that in the stroma. The stromal NADPH levels increased only after 60–90 s of illumination, reached a plateau after 120 s, and dropping quickly (within 30 s) after the light was withdrawn. Unlike the NADH/NAD^+^ ratio in plastid stroma, which dropped below its basal level within 90 s after light withdrawal (Fig. 2e), the stromal NADPH level did not drop to the basal level, even after 4 min of darkness (Fig. 2b). These data imply that the CBB cycle is the major consumer of stromal NADPH, as Rubisco carboxylation stops at ~30 s after light withdrawal when the RuBP pool is exhausted^36^. Since NADP-MDH is active only under light^14,24^, whereas NAD-MDH is active in both light and dark conditions^37^, the malate-OAA shuttle in the chloroplasts can export only stromal NADH reducing equivalents but not stromal NADPH reducing equivalents in darkness (Fig. 2b, e). As the cytosolic NADH/NAD^+^ pool was more oxidized than the stromal pool (Fig. 2d), a continuous export of NADH reducing equivalents, resulting in a stromal NADH/NAD^+^ ratio even lower than that before illumination, was observed (Fig. 2e). All of the above changes were abolished by the application of the photosynthesis inhibitor DCMU (Fig. 2), due to its complete inhibition of the LEF (Supplementary Fig. 10b).

### Photorespiration is the main NADH contributor in mitochondria

**Fig. 3:**
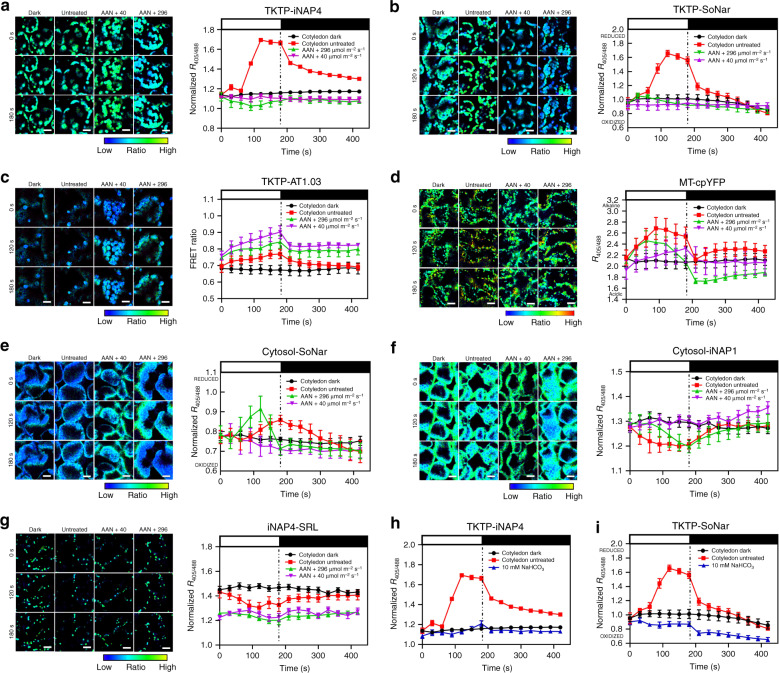
Dynamic changes of NADPH level, NADH/NAD^+^ ratio, MgATP^2-^ ratio, and pH level in 10-day-old seedlings pre-treated with aminoacetonitrile [AAN] or NaHCO_3_. The normalized ratiometric shifts of seedlings pre-treated with 18 mM AAN carrying **a** stromal iNAP4, **b** stromal SoNar, **c** stromal AT1.03, **d** mitochondrial matrix cpYFP, **e** cytosolic SoNar, **f** cytosolic iNAP1, and **g** peroxisomal iNAP4 in cotyledon in response to illumination for an interval of 30–180 s at 296 μmol m^−2^ s^−1^, or 40 μmol m^−2^ s^−1^, are shown. The normalized ratiometric shifts of seedlings in in half-strength Murashige and Skoog medium containing 10 mM NaHCO_3_ carrying **h** stromal iNAP4, **i** stromal SoNar and illuminated at 296 μmol m^−2^ s^−1^ are also shown. The illumination period is indicated by white bars and the values under the black bars represent data obtained after light withdrawal at 180 s (*n* = 5; error bars ± SEM). Scale bars, 20 µm. All iNAP and SoNar results presented were normalized with cytosol-iNAPc, TKTP-iNAPc, and iNAPc-SRL, respectively.

**Fig. 4:**
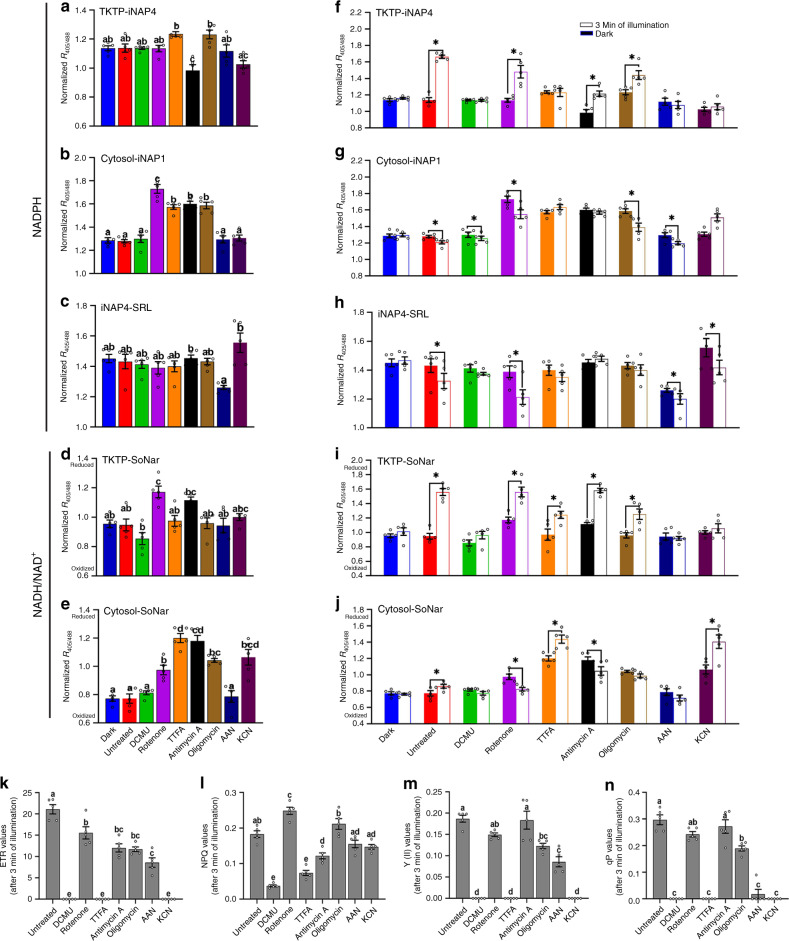
Effects of various inhibitors on the light responses of NADPH levels and NADH/NAD^+^ ratios in 10-day-old seedlings. The inhibitors used were 0.02 mM 3-(3,4-dichlorophenyl)-1,1-dimethylurea (DCMU), 0.05 mM rotenone, 0.1 mM thenoyltrifluoroacetone (TTFA), 0.01 mM antimycin A, 0.01 mM oligomycin; 18 mM aminoacetonitrile (AAN), and 0.5 mM potassium cyanide (KCN). **a**–**e** Normalized *R*_405/488_ ratios of **a** stromal iNAP4, **b** cytosolic iNAP1, **c** peroxisomal iNAP4, **d** stromal SoNar, and **e** cytosolic SoNar. Different letters indicate significant differences as analyzed by Tukey’s HSD test (*P* < 0.05) (*n* = 5; error bars ± SEM). **f**–**j** Ratiometric shifts of normalized *R*_405/488_ ratios of **f** stromal iNAP4, **g** cytosolic iNAP1, **h** peroxisomal iNAP4, **i** stromal SoNar, and **j** cytosolic SoNar in the cotyledon of 10-day-old seedlings in response to illumination at 296 μmol m^−2^ s^−1^ for 180 s are presented as empty bars, while the initial point (0 s) is presented as filled color bars. Asterisks indicate significant differences (*P* < 0.05) before and after 3 min of illuminations as determined by paired *t*-test (*n* = 5; error bars ± SEM). All results were normalized with TKTP-INAPc. **k**–**n** Effects of various inhibitors on **k** electron transport reaction (ETR), **l** non-photochemical quenching (NPQ), **m** effective PS II quantum yield (Y(II)), and **n** coefficient of photochemical quenching (qP). Data from cotyledon in response to illumination for up to 180 s at 280 μmol m^−2^ s^−1^ are presented. Different letters indicate significant differences analyzed by Tukey’s HSD test (*P* < 0.05) (*n* = 5; error bars ± SEM).

Since Arabidopsis GDC mutant is lethal^38^, we used aminoacetonitrile (AAN), an inhibitor of the mitochondrial conversion of glycine to serine and the production of NADH^39^, to investigate the contribution of photorespiration in supplying reducing equivalents. AAN does not affect photosynthesis in protoplasts under non-photorespiratory conditions (high CO_2_), thus supporting its specificity^26^. In the untreated control, illumination at 296 µmol m^−2^ s^−1^ caused a drastic increase in stromal NADPH (Figs. 1b and 2b) and NADH/NAD^+^ ratios (Fig. 2e) but a small decrease in both cytosolic (Figs. 1a and 2a) and peroxisomal NADPH (Figs. 1c and 2c). The cytosolic NADH/NAD^+^ ratio was increased by illumination at 296 µmol m^−2^ s^−1^, but was decreased when the light intensity was only at 40 µmol m^−2^ s^−1^ (Fig. 2d). When AAN was applied, however, even illumination at 296 µmol m^−2^ s^−1^ failed to increase the stromal NADPH pool (Fig. 3a), and the NADH/NAD^+^ ratio (Fig. 3b). By contrast, the production of stromal ATP (Fig. 3c), the electron transport rate (ETR) (Fig. 4k) and the respiration and photosynthesis rates (Supplementary Fig. 10) could still be detected. A pH increment in mitochondrial matrix was still detected in AAN-treated mitochondria (Fig. 3d), indicating that AAN does not interfere with the mETC machinery and gradient formation during electron transport. In addition to inhibitor studies, we further simulated a non-photorespiratory condition by including 10 mM NaHCO_3_ in the half-strength Murashige and Skoog medium during confocal analysis. Similar to the results of AAN treatment, when NaHCO_3_ was applied, illumination at 296 µmol m^−2^ s^−1^ failed to increase the stromal NADPH pool (Fig. 3h), and the NADH/NAD^+^ ratio (Fig. 3i). These results showed that stromal NADPH did not build up when the mitochondria failed to generate NADH by photorespiration.

Under normal conditions, illumination at 296 µmol m^−2^ s^−1^ caused an increase in the cytosolic NADH/NAD^+^ ratio (Fig. 2d) and a decrease in the cytosolic NADPH level (Fig. 2a); however, when photorespiration was prevented by AAN, illumination at 296 µmol m^−2^ s^−1^ transiently increased the cytosolic NADH/NAD^+^ (Fig. 3e), but the decrease in the cytosolic NADPH level became smaller (Fig. 3f). Yet, this effect did not occur at 40 µmol m^−2^ s^−1^ (Fig. 3e, f). Although we were unable to target the SoNar sensor to peroxisomes, we showed that illumination at 296 µmol m^−2^ s^−1^ caused a decrease in NADPH level in peroxisomes (Figs. 1c and 2c). This decrease did not occur under 40 µmol m^−2^ s^−1^ illumination, probably because the rate of photorespiration increased with light intensity. When photorespiration was inhibited by AAN, no decreases in peroxisomal NADPH was seen (Fig. 3g).

Together, these results imply that during photorespiration, a large amount of NADH is generated in the mitochondria through the action of GDC, and concurrently, a large amount of NADH is oxidized in the peroxisomes through the action of HPR. The cytosol is thus in the path of the flow of reducing equivalents between chloroplasts, peroxisomes and mitochondria. The change in the cytosolic NADH/NAD^+^ ratio thus reflects the flow of reducing equivalents between these three organelles.

### Effects of mETC inhibitors on the flow of reducing equivalents

Next, we examined the effects of mETC inhibitors, namely rotenone (complex I), TTFA (complex II), antimycin A (complex III), KCN (cytochrome *c*-dependent respiration), and oligomycin A (ATP synthase), on the NADPH and NADH/NAD^+^ levels in different subcellular compartments (Fig. 4 and Supplementary Fig. 11). The use of rotenone, TTFA, AA, KCN, and oligomycin A increased the cytosolic SoNar ratios, but DCMU and AAN had no effect (Fig. 4, Supplementary Fig. 11). This was also true for cytosolic iNAP1 (except with KCN). This indicates that when the ability of mitochondria to consume NADH in darkness is inhibited, NADH and NADPH accumulate in the cytosol. However, the use of these inhibitors did not increase the basal NADPH level in chloroplasts, indicating that the stromal pool of NADPH in darkness is not affected by mETC activity, probably because stromal NADP-MDH is a light-dependent enzyme. In addition, the basal level of stromal NADH/NAD^+^ ratio was significantly increased by rotenone application, suggesting that reducing equivalents accumulate in the cytosol and stroma after the inhibition of complex I (Fig. 4d).

Upon illumination, an increment in stromal NADPH in seedlings treated with rotenone, AA, and oligomycin A can still be clearly seen (Fig. 4f), as these inhibitors do not inhibit the LEF in chloroplasts. However, TTFA treatment completely suppressed the accumulation of stromal NADPH upon illumination (Fig. 4f) and ETR, Y(II) and qP (Fig. 4k, m, n), probably because TTFA suppresses ferredoxin: NADP^+^ oxidoreductase (FNR)^40^, and reduced the photosynthesis rate (Supplementary Fig. 10b). In addition, we still observed an increase in stromal NADH/NAD^+^ in seedlings treated with rotenone, TTFA and AA upon illumination (Fig. 4i). Surprisingly, rotenone and AA treatments resulted in a decrease in cytosolic NADH/NAD^+^ ratio upon illumination (Fig. 4j), possibly due to de novo NAD^+^ synthesis.

Furthermore, a study with the respiratory inhibitor KCN, as a mean to elucidate the reducing equivalent dynamics in cytochrome *c*-dependent respiration, revealed that KCN treatment also completely inhibited ETR (Fig. 4k). When KCN was applied, the basal cytosolic NADH/NAD^+^ ratio was higher in darkness. In the presence of KCN, whereas illumination did not increase the stromal NAPDH (Fig. 4f) or NADH/NAD^+^ ratio (Fig. 4i), it did increase the cytosolic NAPDH (Fig. 4g) and NADH/NAD^+^ ratio (Fig. 4j).

## Discussion

Monitoring real-time subcellular dynamic changes in reducing equivalents *in planta* has been a long-term challenge. The iNAP and SoNar fluorescence-based sensors were initially introduced in mammalian cells to study the responsiveness of reducing equivalents in homeostasis and cancer cells in vivo^3,4^. SoNar is preferred over another NADH/NAD^+^ sensor, Peredox-mCherry, because it has a 10-fold higher dynamic range and does not aggregate in the cytosol^41^. For the first time, we introduced these iNAP and SoNar sensors into several subcellular compartments in plants (Supplementary Fig. 1). This *in planta* imaging has resolved the bottleneck preventing the study of physiological changes in reducing equivalents at the subcellular level, providing a valuable tool to monitor the real-time dynamics of reducing equivalents without destroying the plant tissues.

We introduced two iNAP sensors with high (iNAP1) and low (iNAP4) NADPH affinities into chloroplasts, peroxisomes and cytosol of Arabidopsis. Our data indicated that iNAP1 is more suitable for observing the changes in cytosolic NADPH levels, whereas iNAP4 is more suitable for monitoring the changes in chloroplast and peroxisome NADPH levels, implying that the cytosolic NADPH concentration may be lower in the cytosol than in the other two compartments. These data corroborate results from past experiments involving the rapid fractionation of illuminated barley protoplasts into chloroplasts and cytosol, in which the total concentrations of NADP^+^ and NADPH were estimated to be 0.6–0.8 mM in chloroplasts and 0.25–0.35 mM in cytosol, when the NADPH/NADP^+^ ratios in these two compartments (1.4 and 1.5) were similar under limiting CO_2_ conditions^42^.

De novo synthesis of NADP^+^ in cytosol, chloroplasts, and peroxisomes is carried out by the ATP-dependent NAD kinases: NADK1, NADK2, and NADK3^43^, among which NADK2 in chloroplasts, is a light-dependent enzyme^44^. Illumination of 3-week-old plants for 5 min to 1 h gradually increases the NADP^+^ and NADPH pools in the rosette leaves of WT plants but not in a *nadk2* mutant, indicating that, under illumination, the synthesis of NADP^+^ from NAD^+^ by NADK2 in the chloroplasts is the major NADP^+^ synthesis pathway. The newly synthesized NADP^+^ is then reduced to NADPH by FNR, and the NADPH/NADP^+^ ratio is maintained at ~1 in the first 5 min of illumination, and gradually decreases to ~0.7 after 60 min of illumination^44^.

By using iNAP sensors with different affinities, we observed not only an increase in stromal NADPH, but also a small decrease of NADPH in the cytosol and peroxisomes upon illumination (Fig. 2). As reported previously from barley leaf protoplast experiments, the stromal NADPH/NADP^+^ ratio declines within 30 s in darkness after illumination^24^; similarly, we observed a rapid decline in stromal NADPH and NADH/NAD^+^ ratio after the light was turned off (Fig. 2). The rapid decline of NADPH level in the first 30 s of darkness can be mainly attributed to the CBB cycle, as Rubisco carboxylation stops ~30 s after light withdrawal^36^. After that, the decline might be mainly due to the malate-OAA shuttle. A small amount of NADPH may also be recycled to NADP^+^ in the light by light-dependent glutathione reductase, but the flux is expected to be small because the substrate, GSSG, is present in the low nanomolar range^35^. Although NAD^+^ can be transported through chloroplasts, mitochondria, and peroxisomes via NDT1, NDT2^45^, and PXN^46^, organelle membranes are impermeable to NADP(H) and NADH due to the lack of transporters. This enables the subcellular compartmentation of NADP(H) and NAD(H) and requires their indirect translocation between subcellular compartments by shuttles, such as the malate-OAA shuttle.

The operation of the malate-OAA shuttle requires the presence of various MDHs in different compartments. Arabidopsis possesses two chloroplastic MDHs, the light-dependent NADP^+^-specific MDH and the light-independent NAD^+^-specific MDH, both of which could transfer stromal surplus reducing equivalents to malate. Our data showed that the post-illumination decrease in stromal NADPH was slower than the drop in stromal NADH/NAD^+^ ratio after 30 s of darkness (Fig. 2), consistent with the natures of these two classes of plastidic MDHs. NADPH is important for fatty acid synthesis and detoxification of oxidative stresses in chloroplasts in the light^47^. This may provide an explanation of why, during evolution, two plastidic MDHs with different substrate specificity and light dependence have been maintained in the plant genomes. During photosynthesis, the surplus NADPH must be exported by the malate-OAA shuttle and therefore requires an active NADP-MDH that functions in a light-dependent manner. In darkness, malate accumulated in the light can be converted to NADH by NAD-MDHs in various subcellular compartments, but not by NADP-MDH to NADPH in plastids.

**Fig. 5:**
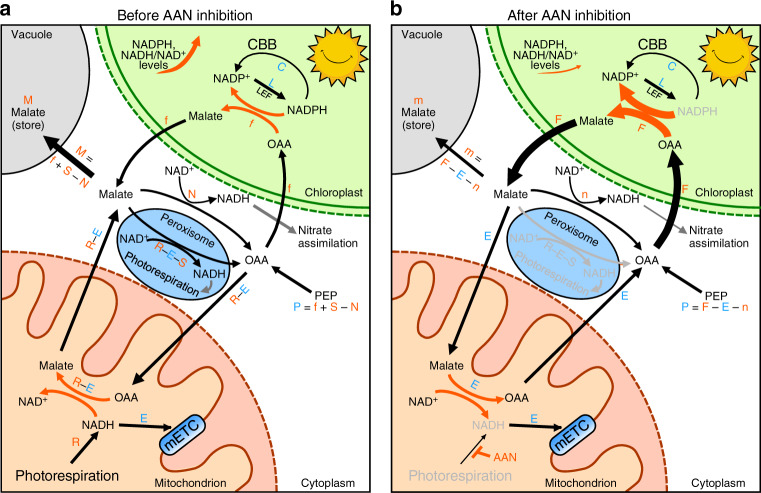
Working model of the flow of malate between several subcellular compartments of photosynthetic C3 plant cells. **a** Malate flow when photorespiration is present. **b** Malate flow when photorespiration is inhibited by AAN or NaHCO_3_. Chloroplast, mitochondrion, and peroxisome are represented by green, brown, and blue circles, respectively. L represents the rate of NADPH production by the LEF and C represents the rate of NADPH consumed by the CBB. Letters in blue (P, C, E, and L) denote entities that are assumed to be unaffected by AAN or NaHCO_3_ treatments at the initial stage of illumination. Letters in red denote entities that are assumed to be affected by AAN or NaHCO_3_ treatments and values that are expected to be larger were represented by capital letters. F is increased but m and n are diminished by AAN treatment.

Fundamental to our study was the question of what serves as the major source of reducing equivalents to mETC during photosynthesis. A key insight from our AAN and NaHCO_3_ experiments was that in the absence of photorespiration, the increases in stromal NADPH and NADH/NAD^+^ upon illumination disappeared (Fig. 3a, b, h, i), suggesting that when photorespiration fails to generate NADH in mitochondria, the mitochondrial malate-OAA shuttle can supply NADH to the mETC (Fig. 3d). This, in turn, could indirectly draw reducing equivalents from the chloroplasts through the cytosolic malate pool and chloroplastic malate-OAA shuttle (Fig. 5). Hence, illumination upon AAN and NaHCO_3_ treatments caused no increase in stromal NADPH (Fig. 3a, h). Our data imply that during photosynthesis, the amount of NADH generated by the photorespiratory GDC is higher than the NADH-dissipating capacity of mitochondria including those of complex I, non-proton-pumping internal NADH dehydrogenases (NDA1 and NDA2), and the mETC and therefore a net export of malate is expected. GDC is the most abundant soluble enzyme in the mitochondria of photosynthetic tissues, and GDC activity increases significantly only after the chloroplasts are mature enough to carry out photosynthesis^48^. Although GDC is highly abundant, the amount of NADH generated by GDC is dependent on the size of the flux of photorespiration cycle, which is dependent on the light intensity. When AAN or NaHCO_3_ were supplied, we detected no increase in stromal NADPH level (Fig. 3a, h) or NADH/NAD^+^ ratio (Fig. 3b, i).

It has been estimated that only 5% of NADPH generated from the LEF in chloroplasts is exported by the malate valve^15^. This implies that when photorespiration does not generate any NADH in mitochondria, in addition to the tricarboxylic acid (TCA) cycle which could supply an unknown amount of reducing equivalents, the mitochondrial ETC can rely on the import of malate for NADH supply. However, under normal photorespiratory conditions, when copious NADH is produced in mitochondria, the peroxisomes could consume NADH at a theoretical ratio of 1:1. In reality, photorespiration is also a source of serine synthesis^49^, and therefore the ratio of mitochondrial NADH production and peroxisomal NADH consumption is >1^8^. As chloroplasts also export malate, it builds up during the day, and most is stored in the vacuole^17^. This indicates that at the whole-cell level, reducing equivalents accumulate during photosynthesis in the form of malate and carbohydrates. At 296 µmol m^−2^ s^−1^ illumination, the cytosolic NADH/NAD^+^ ratio gradually increased (Fig. 2d). Our results (Fig. 2b, e) corroborate the findings of Heineke et al.^50^ indicating that both stromal NADPH and NADH pools increase upon illumination. According to Heineke et al.^50^, the concentrations of malate in spinach chloroplasts and cytosol plus mitochondria during darkness were both calculated as 1 mM, whereas after 8.5 h of illumination, the stromal malate concentration increased to 3 mM but the cytosolic malate concentration was unchanged. The stromal ATP level was also slightly increased upon application of AAN under photorespiratory conditions (Fig. 3c). An increase in ATP/ADP ratio in the presence of AAN was also reported in a protoplast experiment^23^.

Our *in planta* real-time findings are in agreement with previous studies on protoplasts^23,24^ and mitochondria^27^ indicating that photorespiration is the major source of reducing equivalents to the mETC. These reports suggested that the amount of NADH generated by GDC in mitochondria exceeds the capacity of the mETC to dissipate NADH, and therefore surplus NADH generated from photorespiration must be exported to the cytosol from the mitochondria through the malate-OAA shuttle. Several other lines of evidence support this notion. First, plants deficient in mitochondrial MDH grow slowly^51^, and experiments with ^13^C showed an increased labeling of glycine and serine, indicating a limitation of the flux of the photorespiratory pathway^52^. Second, the volume of stroma of spinach mesophyll was calculated as ~20 times the volume of mitochondria^53^. All the 2-PG generated in the chloroplasts has to be cycled into glycine in the peroxisomes, and most has to be converted into serine in mitochondria during photosynthesis. Hence, the GDC activity in mitochondria, representing such a small volume of the whole cell, during photosynthesis must be very robust. Third, GDC is the most abundant soluble protein in the mitochondria of C3 plants, comprising ~40% of the mitochondrial matrix of mature C3 plants^48^. The concentration of the GDC glycine-binding site is estimated at nearly 0.5 mM^54^, and the *k*_cat_ of GDC is on the order of 5–10 per second (10 s^−1^), which is comparable to that of Rubisco (10 s^−1^) but much slower than that of mitochondrial MDH (60 s^−1^ for the forward reaction and 1000–3000 s^−1^ for the reverse reaction)^54^. The reverse reaction of mMDH oxidizes NADH to relieve its inhibition of GDC (*K*_i_ = 15 µM)^55^ and converts OAA to malate to export the surplus NADH from photorespiration to the cytosol.

Various bioenergetic processes driven by illumination, including photosynthesis, photorespiration, fatty acid synthesis, and nitrate assimilation, drive the flux of OAA and malate between the three organelles involved in photorespiration. These three organelles will physically adhere to each other to speed up the flux upon illumination^56^. In plants, the mitochondrion is the major organelle producing cytosolic ATP^2^. The TCA cycle operates in a circular fashion during the dark phase^27,57^: reducing equivalents generated in the TCA cycle that are fed from organic acids like pyruvate and malate^58^ drive the mETC to produce ATP through the mitochondrial ATP synthase^26^. However, during the daytime, the TCA cycle does not run as a circle^27,57^. This could be explained by the presence of surplus NADH generated from GDC, as NADH serves as an inhibitor of the pyruvate dehydrogenase complex, citrate synthase, and 2-oxoglutarate dehydrogenase of the TCA cycle. Taken together, our data extend current knowledge of the flow of reducing equivalents between subcellular compartments to a malate–OAA flux model (Fig. 5a). This model incorporates the following assumptions: (1) The rate of photorespiration and LEF increase with light intensity. Assuming the fixation of CO_2_ and O_2_ occur at a 3:1 ratio^6^. (2) For each O_2_ fixed by Rubisco in the chloroplast, 0.5 molecule of NADH is generated by the GDC in the mitochondria^8^. (3) The capacity of the mETC to dissipate electrons from NADH (E) through Complex I, NDA1, and NDA2 is not instantly affected by the light intensity. (4) The amount of NADH generated from photorespiration (R), at the light intensities we tested, exceeded the capacity of the mETC (E), and the surplus reductants (R–E) are exported by the mitochondrial malate–OAA flux. (5) Some serine generated from photorespiration leaves the photorespiration pathway (S)^9^, and hence the amount of NADH consumed by HPR in the peroxisome is less than the amount of NADH generated by the GDC. (6) Around 5% of electrons generated from the LEF (L) in the form of NADPH are exported by the malate–OAA flux from the chloroplasts (f)^15^. (7) Malate accumulates during photosynthesis is stored in the vacuole. The rate of malate accumulation (M) is f + S − N, where *N* is the amount of NADH consumed by the cytosolic nitrate reductase during nitrogen assimilation. Hence, malate is basically derived from OAA, which is synthesized from PEP (P) and whose input is also equal to f + S − N. (8) Since LEF generates a higher NADPH/ATP ratio than the ratio consumed by the CBB, surplus NADPH is generated during photosynthesis, and though some is exported to extrachloroplastic compartments through the malate–OAA shuttle, a build-up of stromal NADPH occurs (Fig. 2b). After photorespiration is inhibited by AAN or NaHCO_3_ (Fig. 5b), the following assumptions hold: (1) Since no NADH is generated by GDC in the mitochondria (*R* = 0), malate, instead of being exported, is imported into mitochondria to supply NADH to the mETC. (2) As the cytosolic malate/OAA ratio decreases, the malate–OAA flux (F) of the chloroplasts increases and exports reducing equivalents from stromal NADPH at a higher rate. As a result, no increase in NADPH concentration occurs (Fig. 3b). (3) The net synthesis of malate (m) is therefore equal to F – E – n, which is significantly smaller than the M (f + S − N) in the absence of AAN treatment. Hence, the reversible reaction between malate and OAA helps to build up the equilibrium of reducing equivalents between subcellular compartments under light and dark conditions.

## Methods

### Plasmid construction and generation of plant lines

To construct the plant transformation vectors, the cDNAs of iNAP1, iNAP4, iNAPc, and SoNar were amplified with Platinum Pfx polymerase (Invitrogen) from the pcDNA3.1 vectors^3^ and subcloned into the BamHI and XbalI restriction sites of a modified Gateway pENTR/D-TOPO vector (Invitrogen). For targeting to plastids, peroxisomes, and mitochondria, the sequences from *Nicotiana tabacum* chloroplast transketolase transit peptide (TKTP)^59^, peroxisomal targeting signal type 1 (SRL)^60^ or mitochondrial-targeting sequence from *Nicotiana plumbaginifolia* β-ATPase (MT)^31^ were fused to the N-terminus (TKTP and MT) or C-terminus (SRL) of the sensor proteins, respectively. Their unfused versions were used for cytosolic targeting. The cDNAs were then transferred into the recipient plant transformation pEarleyGate100 vector^61^ under the control of the CaMV35S promoter. The pEarleyGate100 vectors were transformed into *Arabidopsis thaliana* plants (ecotype Columbia, Col-0) using the floral dip method^62^. Positive transformants were screened using fluorescence microscopy (Nikon Eclipse 80i). The sensor cDNA sequences were also fused downstream to the 6xHis-tag of the pRSETb vector for protein expression in *Escherichia coli* using the BamHI and HindIII sites. Primer sequences are listed in Supplementary Table 1. All constructs were confirmed by nucleotide sequencing. Furthermore, WT (Col-0) seedlings carrying pH2GW7-MT-cpYFP (mitochondrial matrix pH sensor) and pEarleyGate100-TKTP-AT1.03 (stromal MgATP^2–^ sensor) were as previously reported^2,30,63^.

### Plant cultivation

The transformed plants were germinated and cultivated on plates containing Murashige and Skoog medium^64^ supplemented with 2% (w/v) sucrose and 1% (w/v) Phytagel and stratified for 2 days at 4 °C in darkness before being transferred to a long-day condition with a photoperiod of 16 h at 120–150 µmol photon m^−2^ s^−1^ at 22 °C and 8 h in darkness at 18 °C. Unless otherwise stated, for assay purposes, all seedlings were adapted in darkness for 1 h before image acquisition.

### Recombinant protein expression and purification

*Escherichia coli* BL21 (DE3) pLys cells carrying the pRSETb iNAP1, iNAP4, iNAPc, and SoNar plasmids were grown in 15 ml LB medium containing 100 µg/ml carbenicillin at 37 °C until the cultures reached OD_600_ of 0.6. The expression of His-tagged proteins were induced by 0.1 mM isopropyl β-d-1-thiogalactopyranoside (IPTG), and the cells were then transferred to 18 °C for another 16 h of growth. Cells were collected by centrifugation at 4000×*g* for 30 min at 4 °C, and the cell pellets were suspended in ice-cold buffer A (20 mM sodium phosphate buffer, pH 7.4, containing 0.5 M sodium chloride, 40 mM imidazole, and cOmplete protease inhibitor cocktail (Roche)). After sonication, the lysates were fractionated by centrifugation at 16,000 × *g* for 5 min at 4 °C. The supernatants were loaded into a 1-ml HisTrap^TM^ FF column (GE Healthcare) with a syringe attached to a 0.45-µm filter. After being washed with 2 column volumes of buffer A, proteins were eluted from the resin using buffer B (20 mM sodium phosphate buffer, pH 7.4, containing 0.5 M sodium chloride and 400 mM imidazole). The recombinant proteins were then dialyzed in dialysis buffer (20 mM Tris-HCl, pH 7.5, and 150 mM sodium chloride) overnight and stored at −80 °C before assay. The elutes were separated by 10% (w/v) SDS-PAGE and stained with Coomassie blue dye. NativeMark^TM^ unstained protein standard (Invitrogen) was used as molecular weight marker. Expected molecular masses of 6xHis-tagged iNAP1, iNAP4, and iNAPc proteins were all 42.9 kDa.

### Confocal imaging and ratiometric image analysis

Confocal imaging of seedling was set up as previously described^65^. Imaging was performed with 5× and 40× oil-immersion lenses in multitrack mode using a Zeiss LSM710 NLO confocal microscope (Carl Zeiss Microscopy). Plants expressing iNAP1, iNAP4, and iNAPc in various subcellular compartments were excited sequentially at 405 nm and 488 nm, and emission was detected at 520 ± 16 nm. *R*_iNAP_, *R*_iNAPc_, and *R*_SoNar_ represent the raw ratios of emission excited at 405 nm and 488 nm for iNAP1/4, iNAPc, and SoNar. Autofluorescence was recorded at 431–469 nm and chlorophyll fluorescence was collected at 629–700 nm.

Confocal images were processed with a custom MATLAB-based analysis suite^66^. The ratiometric images were analyzed on a pixel-by-pixel basis using *x*, *y* noise filtering, and fluorescence background subtraction was conducted based on the intensity of the cell samples not expressing sensors from the dark side of the images. Visually, all the ratio profiles in the mesophyll were displayed in pseudocolors. iNAPc is the pH control sensor of iNAP and SoNar^3^. The pH-corrected ratio (normalized *R*_405/488_) was calculated with the formula below,

$${\mathrm{Normalized}}\,R_{\frac{{405}}{{488}}} = R_{{\mathrm{iNAP}}\,{\mathrm{or}}\,{\mathrm{SoNar}}}/R_{{\mathrm{iNAPc}}}$$

### Protoplast isolation and transfection

Isolation of mesophyll protoplasts from the leaves of 28-day-old Arabidopsis plants expressing iNAP4-SRL or iNAPc-SRL was carried out as previously described^67^. The pBI221-mRFP-SRL plasmid was a kind gift from Prof. Liwen Jiang of the Chinese University of Hong Kong. Recombinant plasmids were isolated using MAGEN HiPure Plasmid EF Midi Kit. DNA concentration was adjusted to 600 ng µl^−1^ per 5 kb of DNA. A total of 200 µl protoplast solution was mixed with 10 µl of recombinant plasmid and incubated in darkness at room temperature overnight before acquisition by confocal microscopy.

### In vitro and semi in vivo characterization of iNAP and SoNar

To characterize the sensors, both in vitro and semi in vivo methods were adopted. To determine the in vitro *K*_d_ of the recombinant sensors at room temperature, NADPH, NADP^+^, NADH, and NAD^+^ nucleotides were titrated for iNAP1, iNAP4, iNAPc, and SoNar at pH 7.2, 7.5, 8.1, and 8.4, respectively. Nucleotide concentrations were fixed at 0 µM, 2 µM, 10 µM, 25 µM, 50 µM, 100 µM, 125 µM, 250 µM, 500 µM, and 1000 µM. The purified recombinant sensor proteins were diluted in pseudocytosol medium supplemented with 100 mM potassium gluconate, 30 mM NaCl, 25 mM MES, 25 mM HEPES, 40% sucrose, and 1 mg ml^−1^ (w/v) BSA (pH 7.5) to a final concentration of 0.5 µM. Each assay was performed with 50 μl nucleotides and 50 μl protein arrayed in 96-well black plates (Corning Costar). Fluorescence characteristics of purified iNAP1, iNAP4, iNAPc, and SoNar were detected by a multimode reader (Victor X3, PerkinElmer) with sequential dual excitation at 405 nm and 485 nm, whereas emission was detected at 520 nm.

For semi in vivo experiment, 6–7-day-old seedlings were infiltrated with 100 µM digitonin buffer supplemented with 100 µM digitonin, 100 mM potassium gluconate, 1 mM MgCl_2_, 5 mM EGTA, and 10 mM HEPES, and pH 7.5. Seedlings containing cytosolic sensors were then infiltrated in the buffer with different NADPH concentrations (0 µM, 0.1 µM, 1.0 µM, 4.0 µM, 8.0 µM, 10.0 µM, 25.0 µM, 50.0 µM, 100.0 µM, and 500.0 µM for iNAP) or a total concentration of 100 µM NADH/NAD^+^ of different ratios (for SoNar) in pseudocytosol medium for 5 min before imaging.

### Responses of sensors to oxidants, reductant, and antioxidants

To obtain the potential redox ratios of the sensor lines in vitro and *in planta*, purified iNAP or SoNar recombinant proteins and 10-day-old seedlings were either treated with 10 mM dithiothreitol (DTT) or oxidized with 10 mM H_2_O_2_ or 30 µM menadione. 10 mM l-ascorbic acid or 10 mM reduced glutathione were also tested via in vitro study. Purified recombinant proteins were diluted in pseudocytosol medium as described above. Seedlings were infiltrated for 5 min in half-strength Murashige and Skoog medium with or without reagents before imaging.

### Light intensity analysis

Seedlings were cultivated as described above. To explore the effect of light intensity, seedlings were exposed to different light intensities (40 µmol m^−2^ s^−1^ or 296 µmol m^−2^ s^−1^ using confocal microscope equipped with a halogen lamp (HAL 100 W; Philips). The light intensity of confocal microscope halogen lamp (HAL 100 W; Philips) was determined using a Lutron LX-120 light meter (Lutron, Taipei, Taiwan) and converted into µmol m^−2^ s^−1^ using the formula as previously described^68^. Each step took 30 s of illumination and imaging was immediately captured after each illumination period. After 3 min with 30 s intervals of exposure to the light, relaxation of seedlings in darkness was monitored for 4 min, during which the images were acquired every 30 s. Light intensity response curves for compartmentalized iNAP1 and iNAP4 were normalized by iNAPc.

### Inhibitors treatment

Ten-day-old seedlings were infiltrated for 5 min in half-strength Murashige and Skoog medium with or without inhibitors. The following inhibitors were prepared and used to treat the seedlings in this study: inhibitors of photosynthesis (0.02 mM DCMU), mitochondrial complex I (0.05 mM rotenone), SDH (0.1 mM TTFA), complex III (0.01 mM antimycin A), complex IV (0.5 mM KCN), mitochondrial ATP synthase (0.01 mM oligomycin A)^2^, and photorespiration (18 mM AAN^69^ and 10 mM NaHCO_3_^70^). Unless otherwise stated, after infiltration, all seedlings were incubated in the dark for 1 h before imaging. The 10-day-old pretreated seedlings were illuminated at 296 µmol m^−2^ s^−1^.

### Determination of oxygen consumption rate at room temperature

Respiratory oxygen consumption and photosynthetic oxygen evolution rate were measured with a Clark-type electrode (Oxygraph plus, Hansatech). Measurements were performed with 0.025 g of 10-day-old seedlings. Following an initial dark equilibration of the seedlings, oxygen was measured in the dark for 4 min before the seedlings were illuminated at an intensity of 296 µmol m^−2^ s^−1^ for 3 min at room temperature and then subjected to 3 min of darkness. The consumption or evolution rate was determined for each minute interval. The oxygen levels from the fifth to sixth and eighth to ninth minutes were used to calculate the evolution rate and consumption rate (nanomoles of O_2_ per second), respectively. The rate of photosynthesis was calculated as the evolution rate minus consumption rate. The respiration rate (%) was calculated as follows,

$${\mathrm{Respiration}}\,{\mathrm{rate}}\,({\mathrm{\% }}) = \frac{{\frac{{{\mathrm{oxygen}}\,{\mathrm{consumption}}\,{\mathrm{rate}}\,({\mathrm{treated}}\,{\mathrm{seedlings}})}}{{{\mathrm{total}}\,{\mathrm{seedling}}\,{\mathrm{fresh}}\,{\mathrm{weight}}\,({\mathrm{g}})}}}}{{{\mathrm{oxygen}}\,{\mathrm{consumption}}\,{\mathrm{rate}}\,({\mathrm{untreated}}\,{\mathrm{seedlings}})}} \times 100$$

### Determination of chlorophyll fluorescence parameters

Fluorescence parameters were measured with a chlorophyll fluorometer (MAXIPAM, Heinz Walz) at room temperature. Treated seedlings were incubated in darkness for 1 h. The fluorescence yield was then measured with actinic light at 280 µmol m^−2^ s^−1^ at interval of 30 s up to 3 min in the presence or absence of inhibitors, and following by 4 min of darkness. The effective quantum yield (Y(II)), non-photochemical quenching (NPQ), and photochemical quenching (qP) were calculated as [(*F*_m_′−*F*_s_)*/F*_m_′], [(*F*_m_−*F*_m_′)/*F*_m_′], and [(*F*_m_′−*F*_s_)/(*F*_m_′−*F*_o_)], respectively.

### Data analysis

All data are shown as the means with standard error (mean ± SEM). The collected data were analyzed for statistical significance using analysis of variance (Tukey’s HSD) and paired *t*-test at *P* < 0.001, *P* < 0.01, and *P* < 0.05 by SPSS (Version 20).

### Reporting summary

Further information on research design is available in the Nature Research Reporting Summary linked to this article.

## Supplementary information

Supplementary Information

Reporting Summary

## Data Availability

We declare that the main data supporting the findings of this study are available within the article and its Supplementary Information files. Source data are provided with this paper. Extra data are available from the corresponding author upon request.

## Source data

Source Data
