# Supplementary material for: In planta study of photosynthesis and photorespiration using NADPH and NADH/NAD+ fluorescent protein sensors
Source: Nat Commun. 2020 Jun 26;11:3238. doi: 10.1038/s41467-020-17056-0 (PMC7320160; doi:10.1038/s41467-020-17056-0)
Supplement: Supplementary file 1 — Supplementary Information [file 41467_2020_17056_MOESM1_ESM.pdf]

Supplementary Information for

In planta study of photosynthesis and photorespiration using NADPH and NADH/NAD$^+$ fluorescent protein sensors

Lim *et al.*

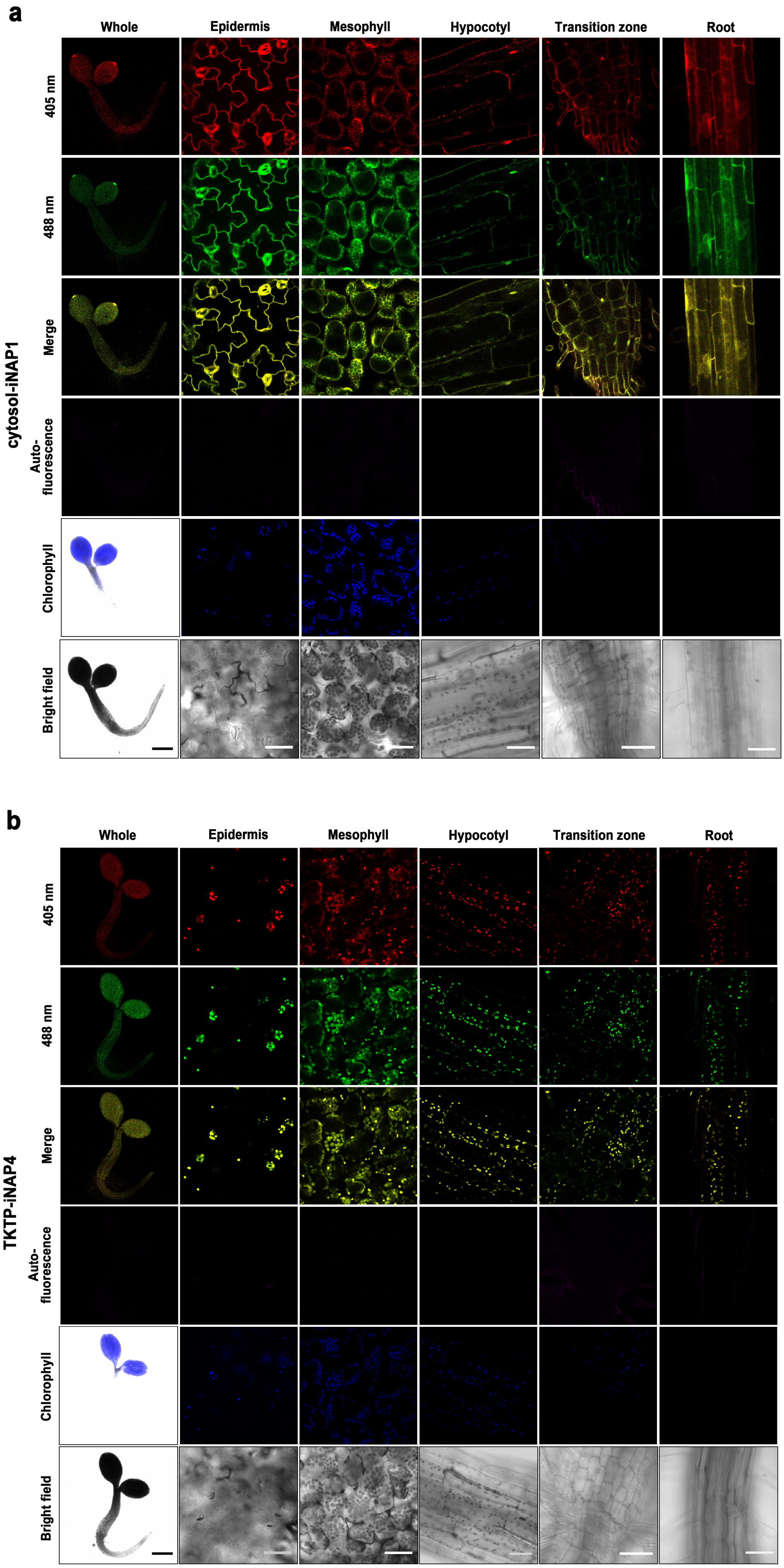

a
Whole
Epidermis
Mesophyll
Hypocotyl
Transition zone
Root
405 nm
488 nm
Merge
Auto-fluorescence
Chlorophyll
Bright field
cytosol-iNAP1
b
Whole
Epidermis
Mesophyll
Hypocotyl
Transition zone
Root
405 nm
488 nm
Merge
Auto-fluorescence
Chlorophyll
Bright field
TKTP-iNAP4

c

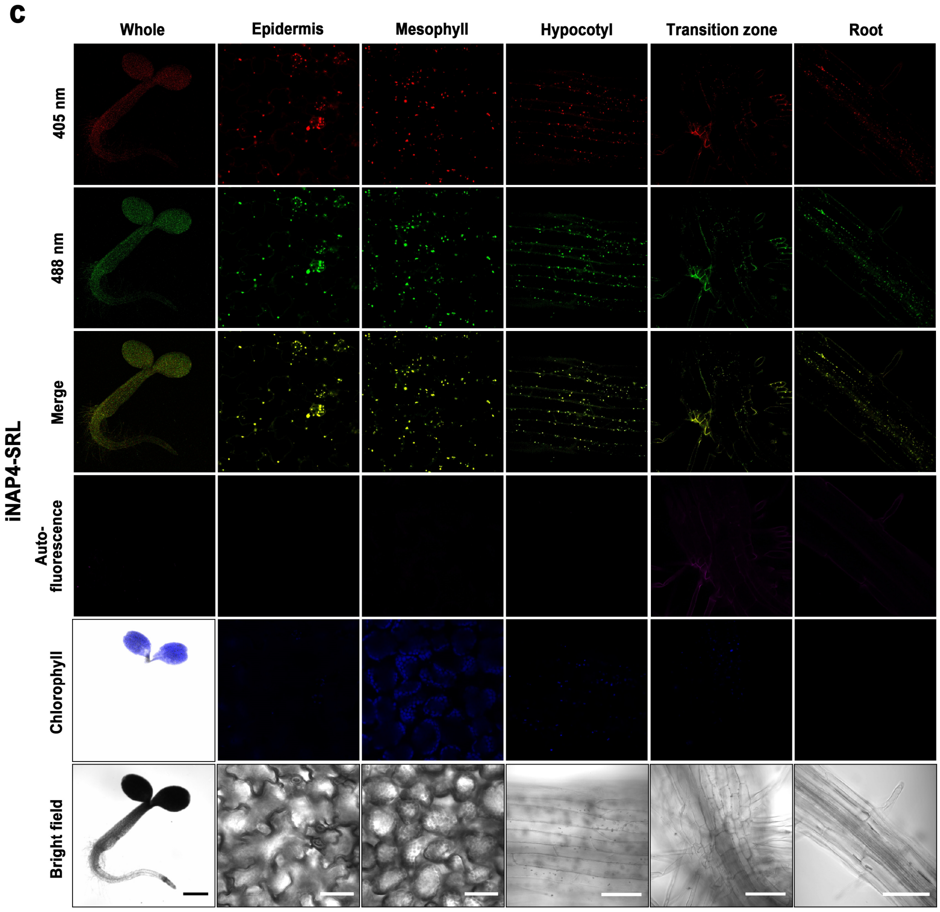

Whole
Epidermis
Mesophyll
Hypocotyl
Transition zone
Root
iNAP4-SRL
405 nm
488 nm
Merge
Auto-fluorescence
Chlorophyll
Bright field

d

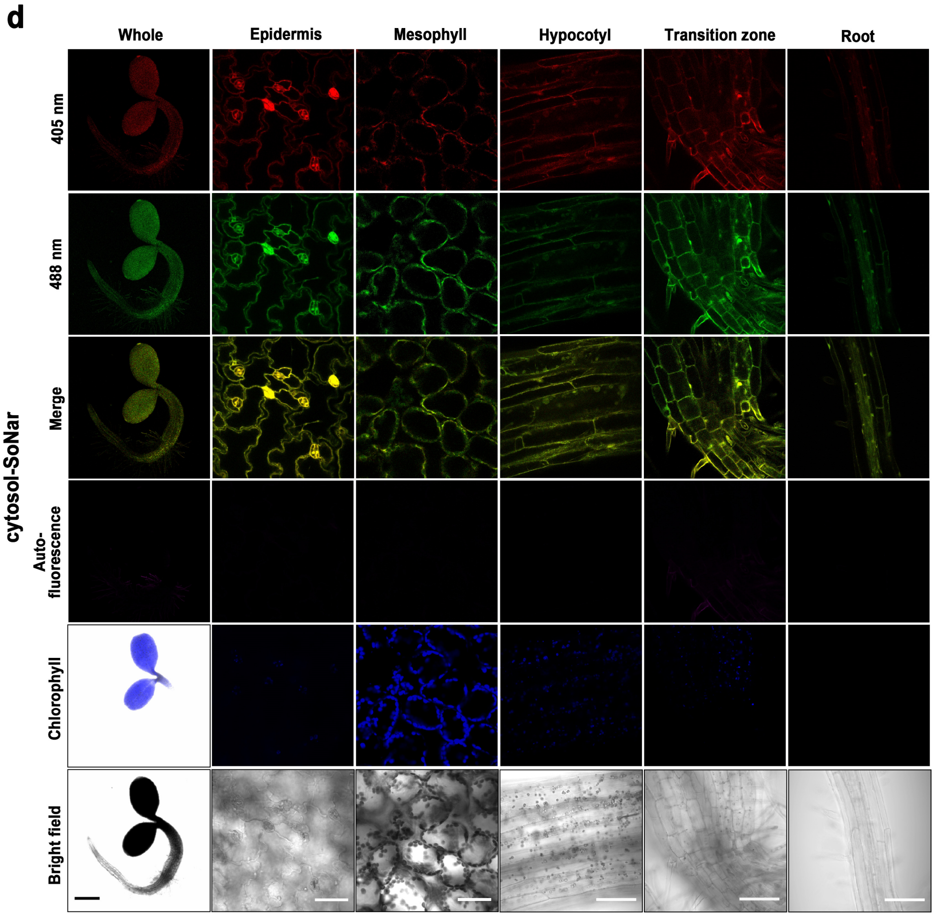

Whole
Epidermis
Mesophyll
Hypocotyl
Transition zone
Root
cytosol-SoNar
405 nm
488 nm
Merge
Auto-fluorescence
Chlorophyll
Bright field

e

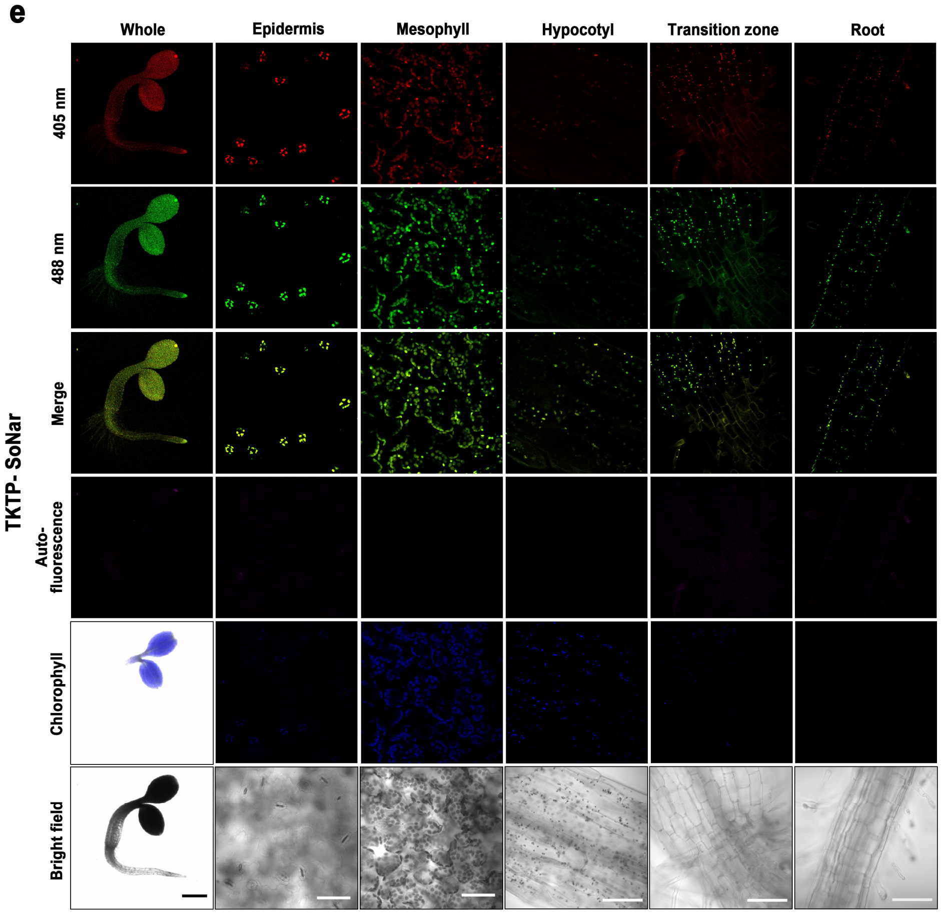
Whole
Epidermis
Mesophyll
Hypocotyl
Transition zone
Root
405 nm
488 nm
Merge
Auto-fluorescence
Chlorophyll
Bright field
TKTP- SoNar

f

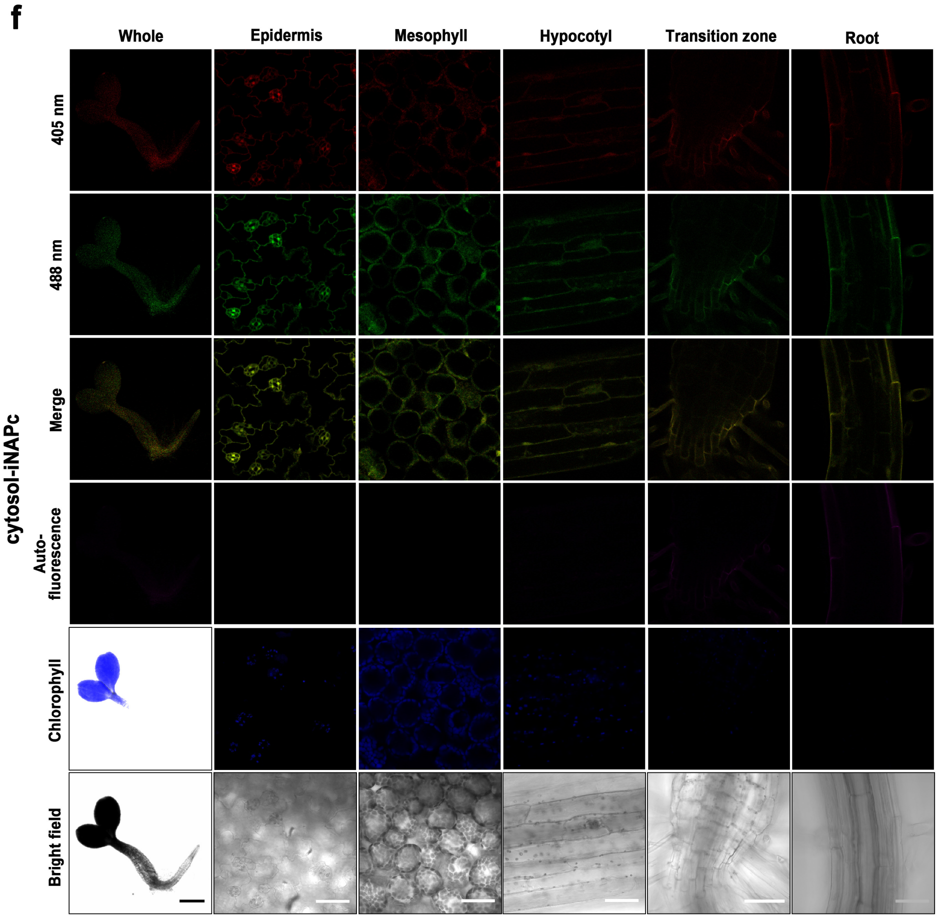
Whole
Epidermis
Mesophyll
Hypocotyl
Transition zone
Root
405 nm
488 nm
Merge
Auto-fluorescence
Chlorophyll
Bright field
cytosol-iNAPc

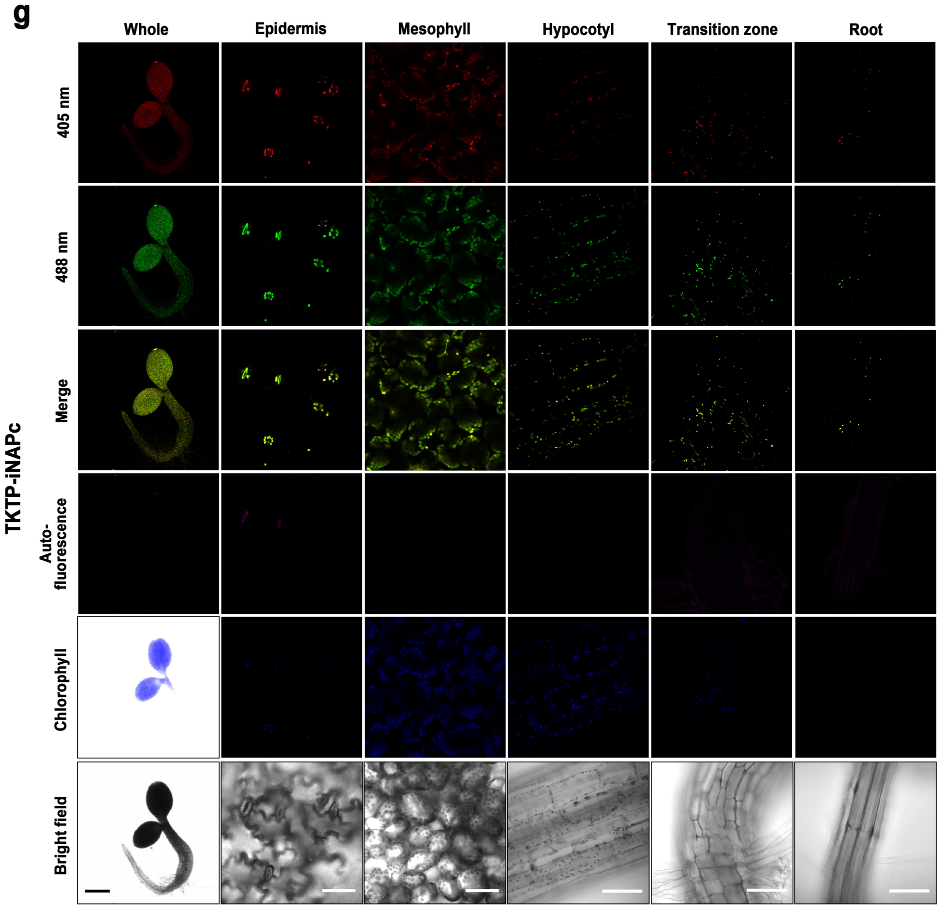
g
Whole
Epidermis
Mesophyll
Hypocotyl
Transition zone
Root
405 nm
488 nm
Merge
Auto-fluorescence
Chlorophyll
Bright field
TKTP-iNAPc

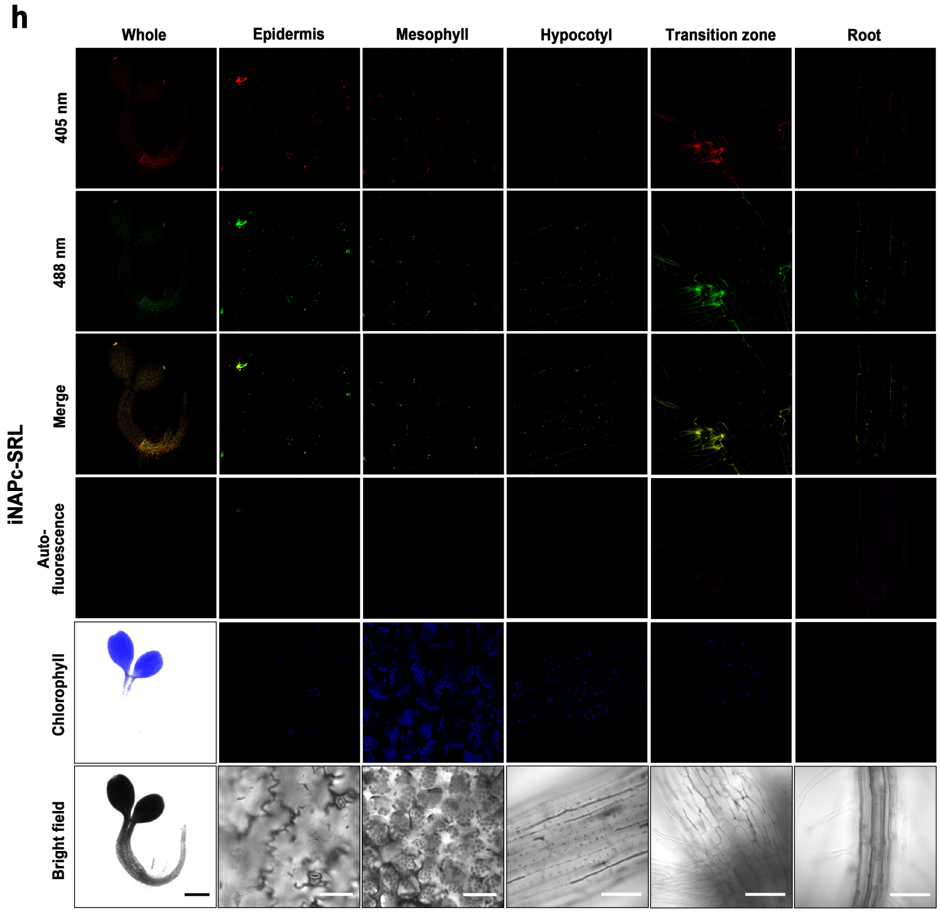
h
Whole
Epidermis
Mesophyll
Hypocotyl
Transition zone
Root
405 nm
488 nm
Merge
Auto-fluorescence
Chlorophyll
Bright field
iNAPc-SRL

i

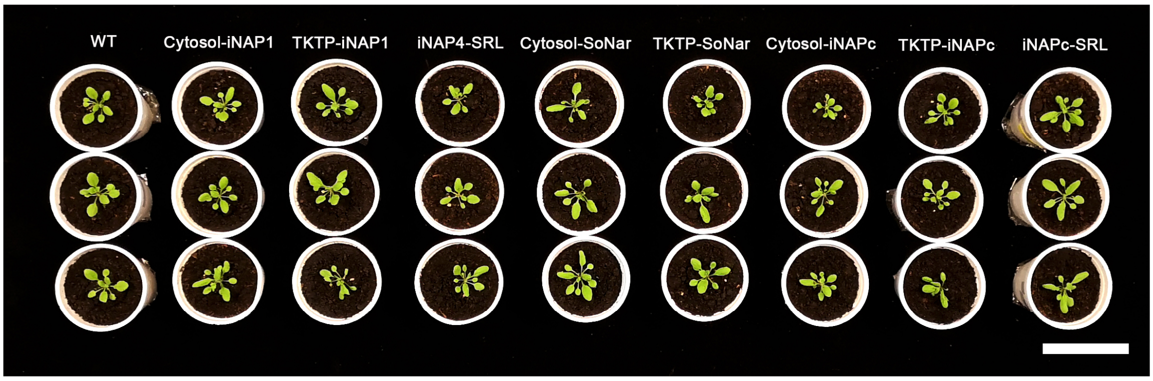

j

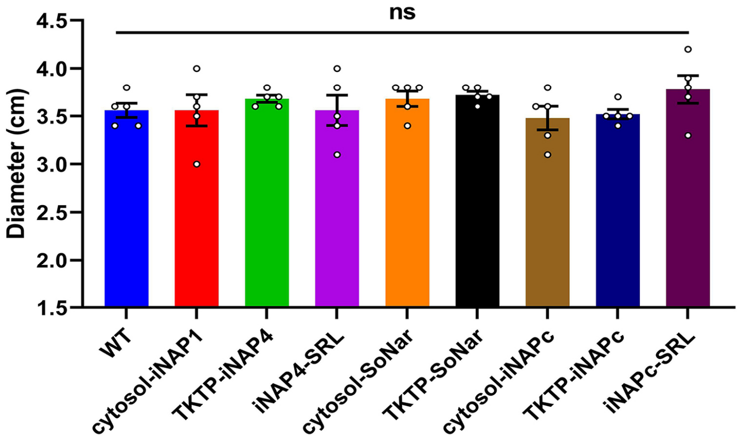

Supplementary Figure 1. Overview of the images of various tissues of 3-d-old (whole seedlings) and 6-d-old (plant tissue) seedlings expressing various sensors. (**a-c**) The NADPH sensors **a**) cytosol-iNAP1, **b**) TKTP-iNAP4 and **c**) iNAP4-SRL. (**d, e**) The NADH/NAD$^+$ sensors **d**) cytosol-SoNar and **e)** TKTP-SoNar. (**f-h**) The controls **f**) cytosol-iNAPc, **g**) TKTP-iNAPc and **h**) iNAPc-SRL. Sensors were plotted as pseudocolours in individual channels. 405 nm represents excitation at 405 nm with emission at 520 ± 16 nm (red); 488 nm represents excitation at 488 nm with emission at 520 ± 16 nm (blue); 'merge' indicates the overlay of the above two images. Autofluorescence was recorded with excitation wavelength of 405 nm and emission at 450 ± 19 nm (magenta), chlorophyll fluorescence was recorded with excitation at 488 nm and emission at 627-700 nm (blue). Black scale bars, 500 µm; white scale bars, 50 µm. **i**) Phenotype of 27-d-old plants of different lines (cytosol-iNAP1, TKTP-iNAP4, iNAP4-SRL, cytosol-SoNar, TKTP-SoNar, cytosol-iNAPc, TKTP-iNAPc and iNAPc-SRL) were compared with that of the wild type (Col-0), seedlings. White scale bar, 8 cm. **j**) Plant diameters of the 27-d-old plants were measured, no significant difference as determined by Tukey's HSD ($P < 0.05$); $n = 5$; error bars ± SEM; ns, no significant difference.

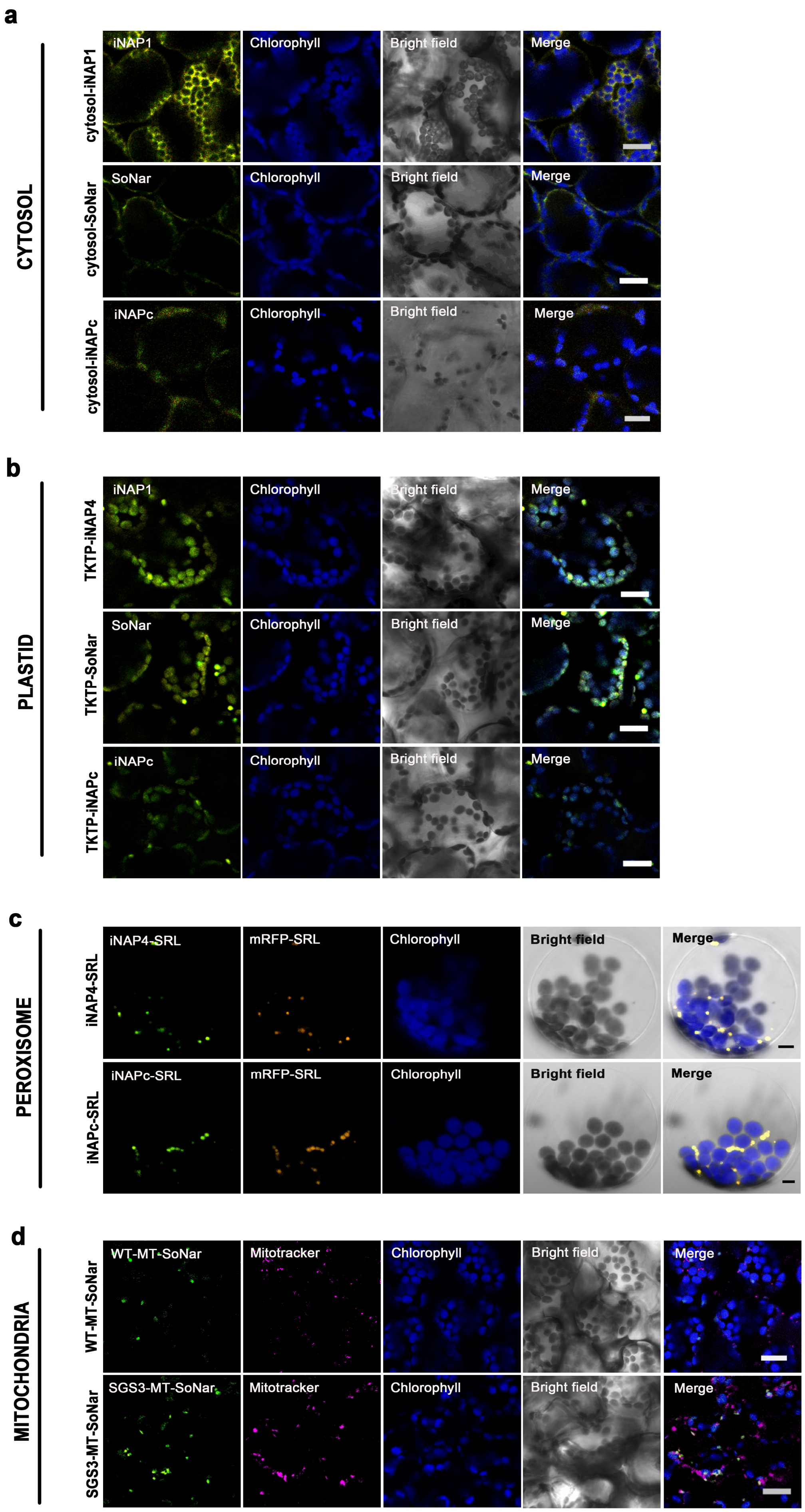

a
CYTOSOL
cytosol-iNAP1
cytosol-SoNar
cytosol-iNAPc
iNAP1
SoNar
iNAPc
Chlorophyll
Bright field
Merge
b
PLASTID
TKTP-iNAP4
TKTP-SoNar
TKTP-iNAPc
c
PEROXISOME
iNAP4-SRL
iNAPc-SRL
mRFP-SRL
d
MITOCHONDRIA
WT-MT-SoNar
SGS3-MT-SoNar
Mitotracker

Supplementary Figure 2. **Verification of subcellular localization of NADPH (iNAP), NADH/NAD$^+$ (SoNar) and control (iNAPc) sensor lines**. Subcellular localization of iNAP sensors in **a**) cytosol **b**) plastid stroma of mesophyll of 20-d-old plants and **c**) peroxisome of protoplasts isolated from 28-d-old plants were confirmed by fluorescence imaging using confocal microscopy (dual excitation at 405 nm and 488 nm, and emission at 520 ±15 nm for iNAP and SoNar; 629-700 nm for chlorophyll auto-fluorescence; and excitation at 543 nm and emission at 580 ± 15 nm for mRFP and mitotracker). **d)** The mitochondrial targeting presequence failed to direct the SoNar sensor to the mitochondria as the sensor signals did not overlap with the signals of mitotracker. No transformants with iNAP signals were obtained after repeated transformation. The images were plotted as pseudocolour. White scale bar, 20 µm, black scale bar, 5µm.

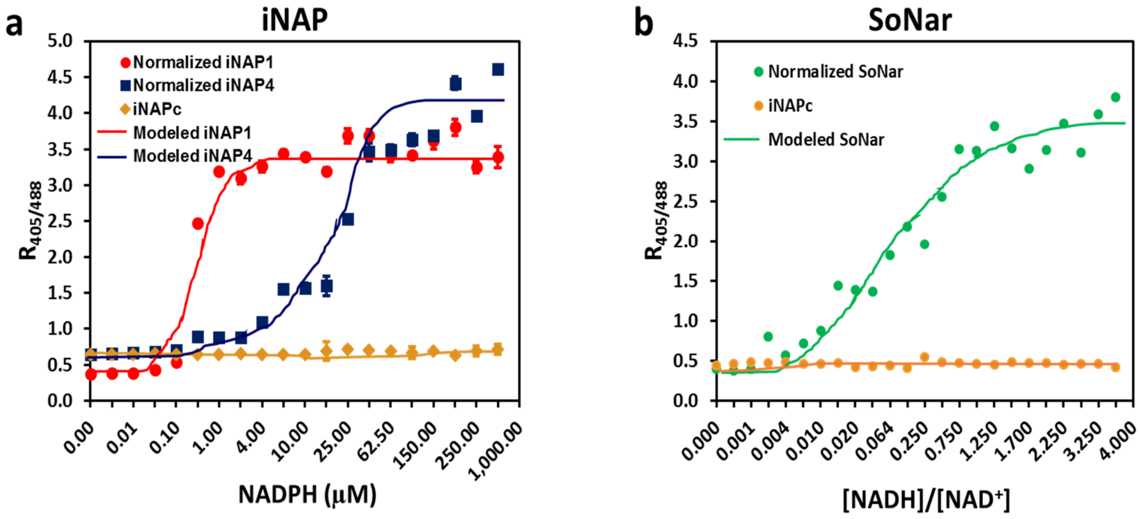

Supplementary Figure 3. ***In vitro* titration**. **a**) NADPH titration curves of recombinant iNAP1 and iNAP4. The proteins were excited at 405 nm and 485 nm in a fluorescence plate reader and the ratios of their emissions at 520 nm were normalized to the iNAPc data. The readings were taken at room temperature. Modeled calibration curves were calculated using dissociation kinetics to determine the sensors' K_d for NADPH. *In vitro* K_d of iNAP1 and iNAP4 were determined to be 0.29 µM and 30 µM, respectively. **b**) NADH/NAD^+ titration curve of normalized data of SoNar excited at 405 nm and 485 nm in a fluorescence plate reader at room temperature (*n* =3; error bars ± SEM). The *in vitro* K_d of SoNar was determined to be 0.036.

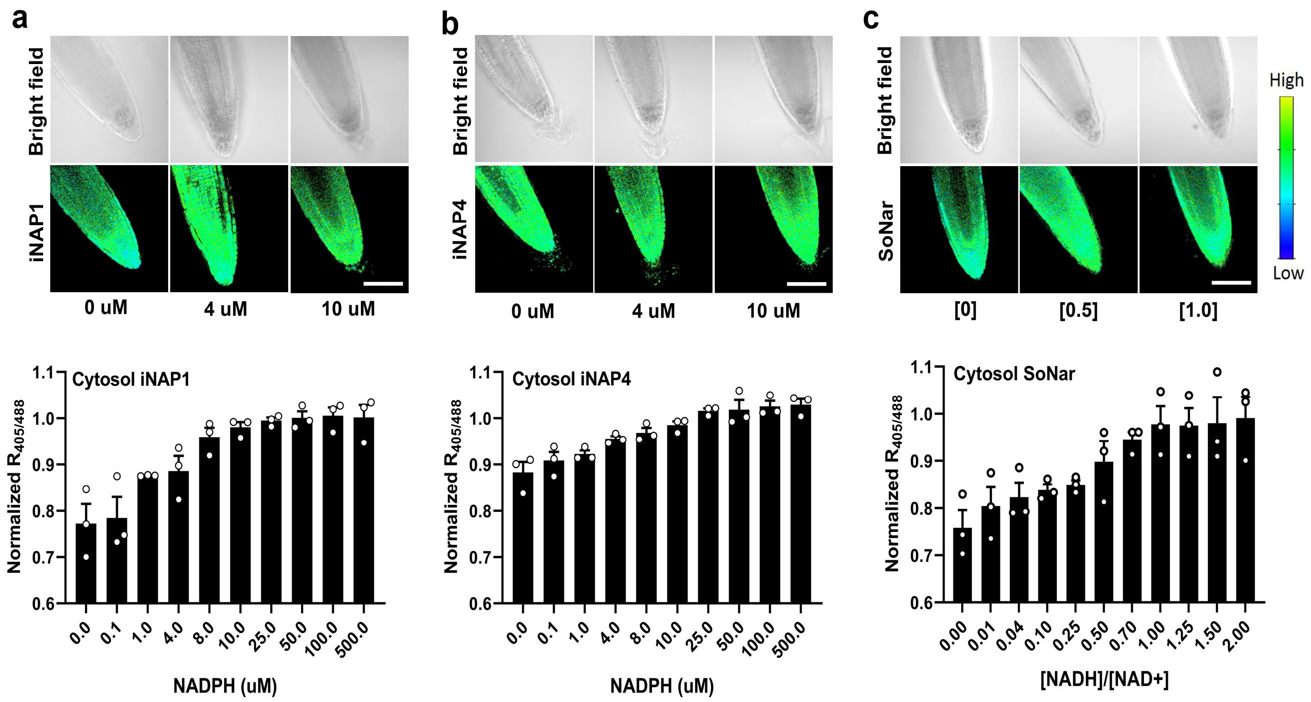

Supplementary Figure 4. The semi *in vivo* responses of the sensors in seedling root tips to various levels of exogenous NADPH or NADH/NAD$^+$. Changes in normalized ratios were observed in 6 and 7-d-old seedling root tips expressing **a**) cytosol iNAP1, **b**) cytosol iNAP4 and **c**) cytosol SoNar. Scale bar, 100 µm; *n* = 3; error bars ± SEM. Calibration curves were calculated using dissociation kinetics to determine the sensors' K_d for NADPH and NADH/NAD$^+$.

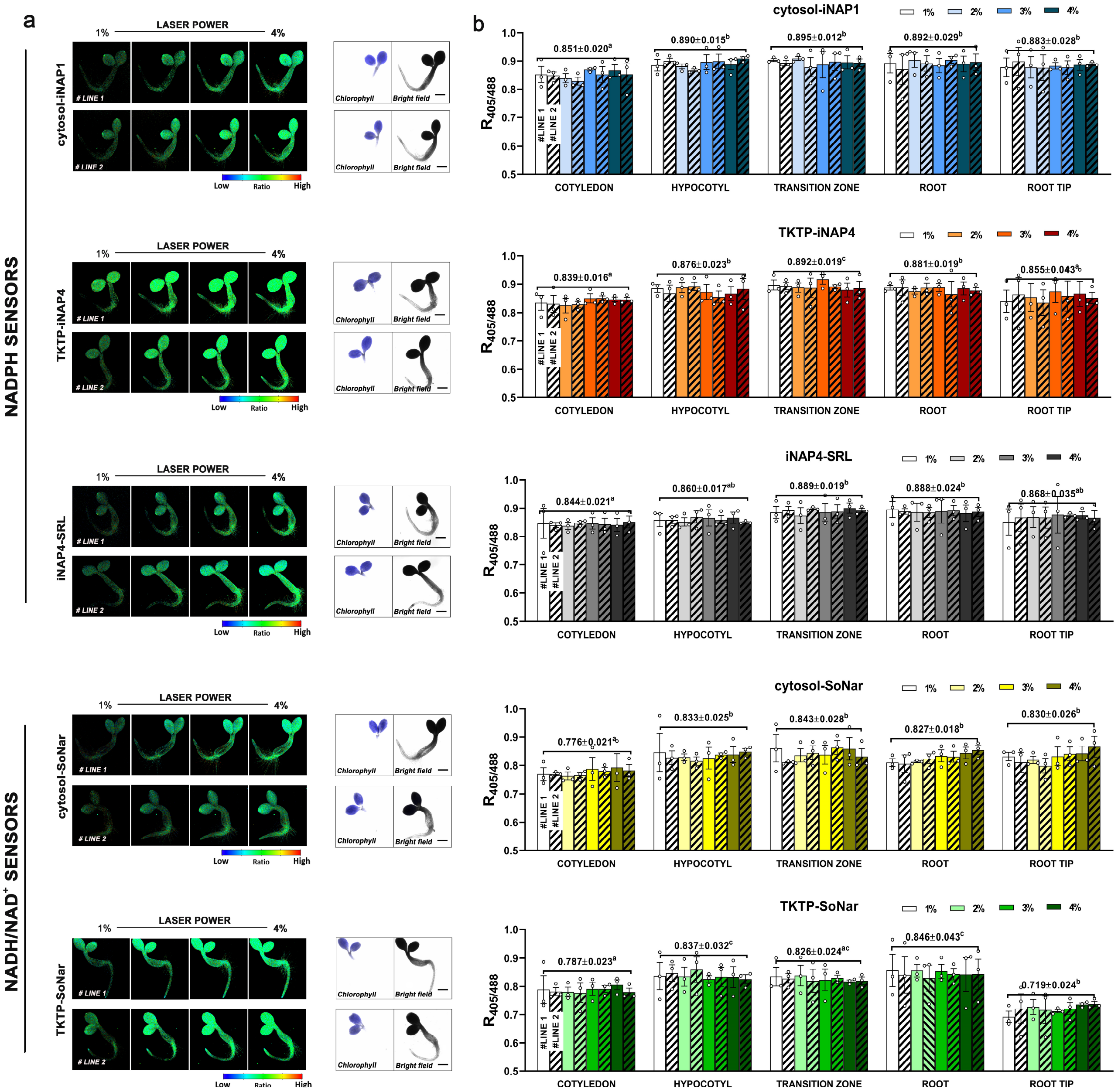

Supplementary Figure 5. **Raw $R_{405/488}$ ratios obtained from 3-d-old seedlings of two independent iNAP (cytosol, TKTP and SRL) and SoNar (cytosol and TKTP) sensor lines with different confocal laser powers (1%, 2%, 3%, and 4%). a**) Fluorescence from dual excitation wavelengths (sequential excitation at 405 nm and 488 nm; emission at 520 ± 16 nm for iNAP; excitation at 488 nm and emission at 629–700 nm for chlorophyll autofluorescence) were recorded with a gradual increase of confocal laser power from 1% to 4%. Ratio images are presented in pseudocolor where high $R_{405/488}$ (red) correspond to high NADPH levels. Scale bar, 500 µm. **b**) Graphs represent the data of two independent lines expressing the same sensor ($n = 3$; error bars ± SEM). Within each compartment, there were no statistical differences between different confocal laser power levels; tissues with significant differences as determined by Tukey's HSD ($P < 0.05$) ($n = 24$) are indicated with different letters.

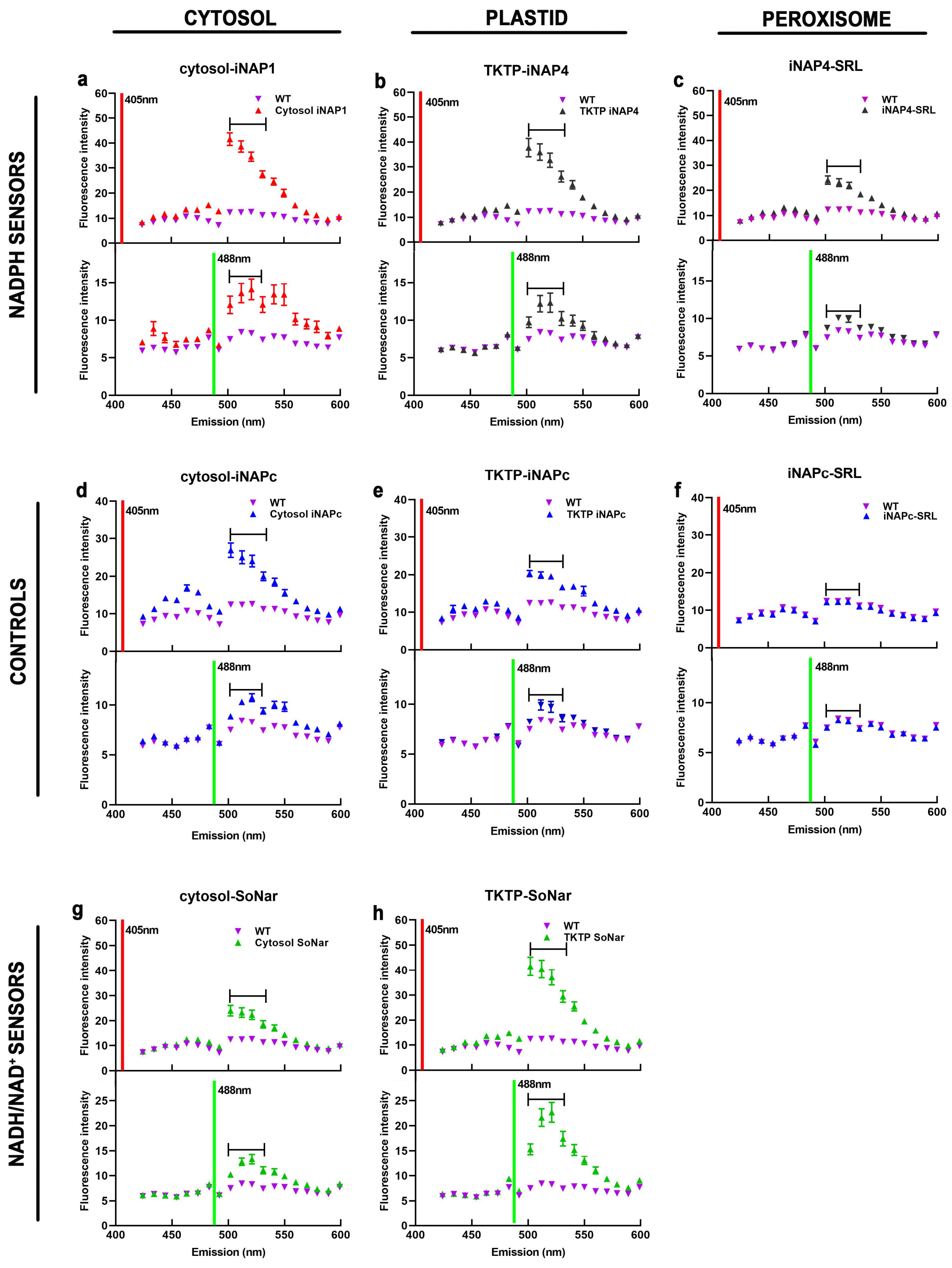

Supplementary Figure 6. **Emission spectra of 10-d-old seedlings expressing different sensors. a**) cytosol-iNAP1, **b**) TKTP-iNAP4, **c**) iNAP4-SRL, **d**) cytosol-iNAPc, **e**) TKTP-iNAPc, **f**) iNAPc-SRL, **g**) cytosol-SoNar, **h**) TKTP-SoNar, and no sensor (WT) were recorded with a 405 nm (red line) and a 488 nm (green line) excitation wavelength. The emission was collected from 424 nm to 599 nm with a resolution of 9.6nm (n = 10; errors bar ± SEM). Black bar indicates the collection range of emission wavelength throughout this study.

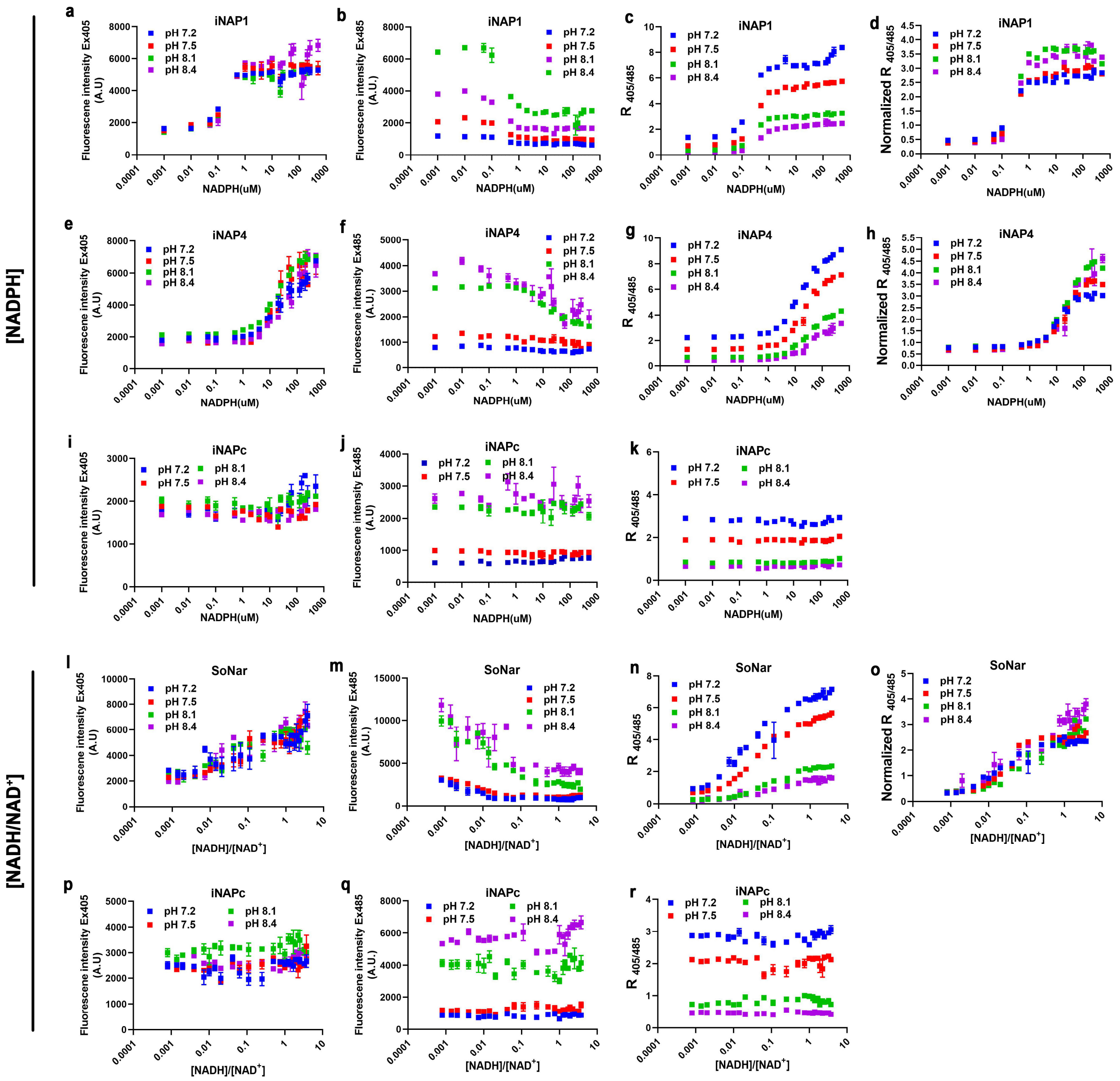

Supplementary Figure 7. Determination of fluorescence intensities of recombinant iNAP1, iNAP4, iNapc and SoNar with excitation at 405 nm or 485 nm and emission at 520 nm. The readings at the indicated pH (7.2, 7.5, 8.1, 8.4) and various pyridine nucleotide concentrations were measured *in vitro* with a fluorescence plate reader. **a-b**) NADPH titration curves of iNAP1 when excited at 405 nm or 485 nm at the indicated pH. **c**) NADPH titration curve of iNAP1 $R_{405/485}$ at the indicated pH. **d**) NADPH titration curves of iNAP1 at various pHs after normalization with iNAPc. **e-f**) NADPH titration curves for iNAP4 when excited at 405 nm or 485 nm at the indicated pH. **g**) $R_{405/485}$ of iNAP4. **h**) NADPH titration curves of iNAP4 at various pHs after normalization with iNAPc. **i-j**) NADPH titration curves for iNAPc with excitation of 405 nm or 485 nm. **k**) $R_{405/485}$ of iNAPc. **i-m**) Response of SoNar excited with 405 nm or 485 nm at different pHs (7.2, 7.5, 8.1, 8.4) against various concentrations of NADH/NAD^+; the total pyridine nucleotide concentration was 100 µM. **n**) $R_{405/485}$ of SoNar. **o**) NADH/NAD^+ titration curves of SoNar at various pH after normalization with iNAPc. **p-q**) Response of iNAPc excited with 405 nm or 485 nm at different pH (7.2, 7.5, 8.1, 8.4) against various NADH/NAD^+ ratios; the total pyridine nucleotide concentration was 100 µM. **r**) 405/485 nm ratio of iNAPc, *n* =3; error bars ± SEM; A.U., arbitrary units.

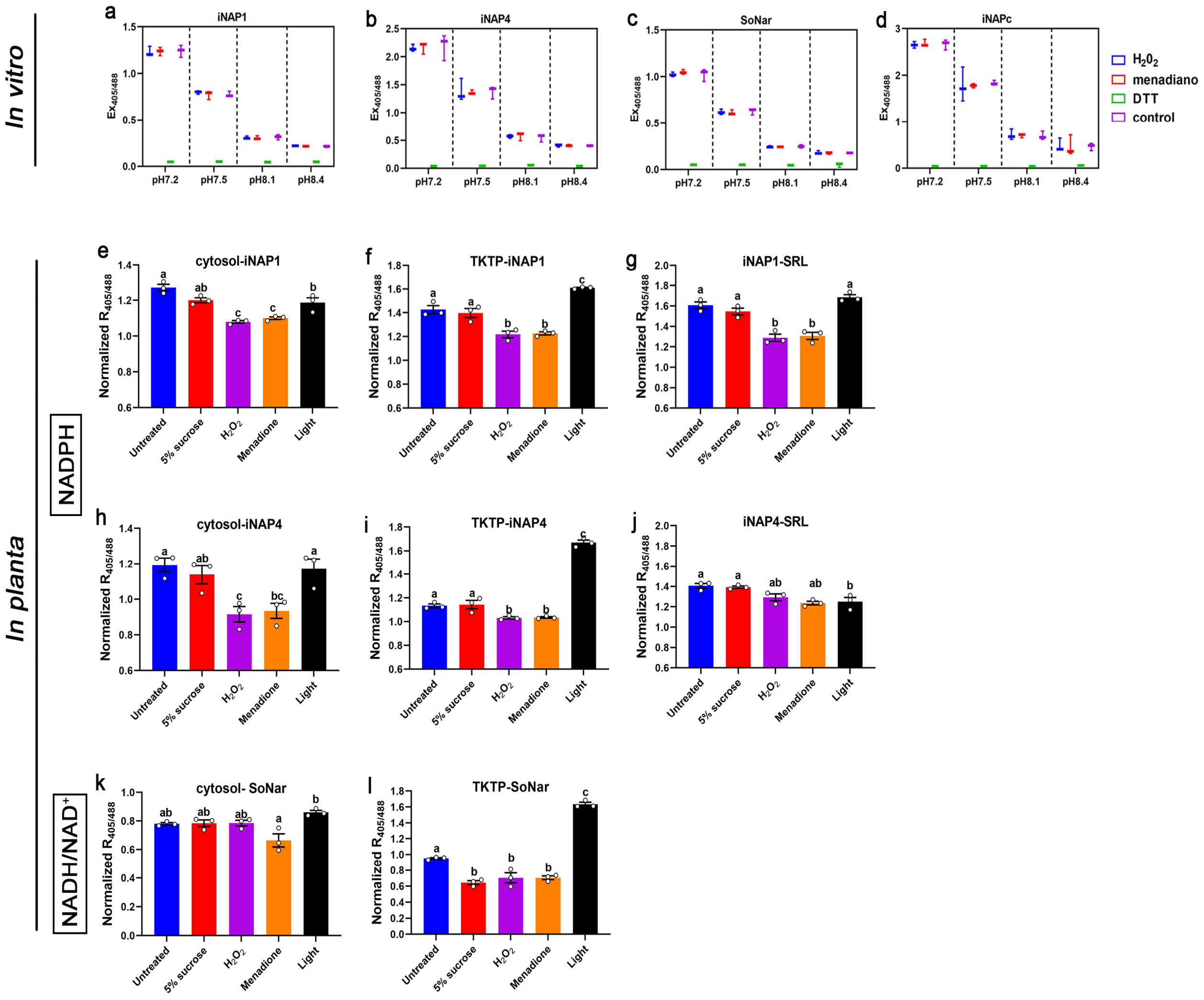

Supplementary Figure 8. **Ranges of *in vitro* and *in planta* sensor ratios treated with various oxidizing and reducing agents.** *In vitro* monitoring of **a**) iNAP1, **b**) iNAP4, **c**) SoNar and **d**) iNAPc in buffers with different pH and 10 mM DTT, 10 mM H_2O_2 or 30 µM menadione, respectively, in a multiwell fluorescence plate reader. $n = 3$; error bars ± maximum and minimal ratio. The effects of 10 mM H_2O_2 and 30 µM menadione treatments on the sensor ratios in various compartments of cotyledon mesophyll of 10-d-old seedlings were presented in **e**) cytosol-iNAP1, **f**) plastid stromal iNAP1, **g**) peroxisomal iNAP1, **h**) cytosol-iNAP4, **i**) plastid stromal iNAP4, **j**) peroxisomal iNAP4, **k**) cytosol SoNar and **l**) plastid stromal SoNar. $n = 3$; error bars ± SEM. Treatments with significant differences as analyzed by Tukey's HSD ($P < 0.05$) are indicated with different letters.

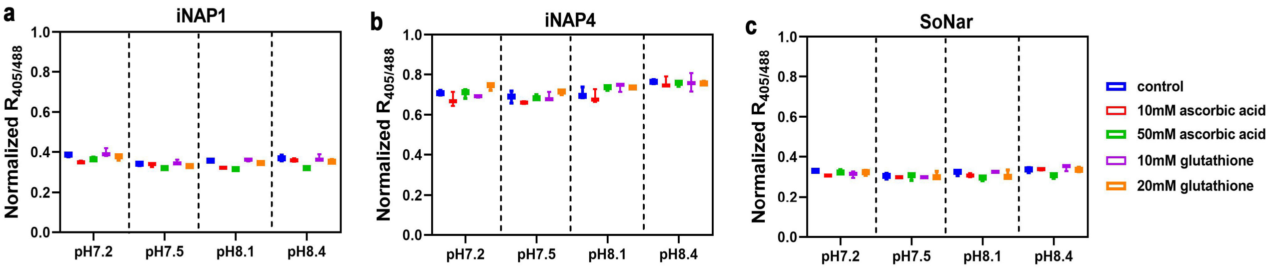

Supplementary Figure 9. The $R_{405/480}$ of recombinant sensors are not affected by L-ascorbate and reduced glutathione. Normalized **a**) iNAP1, **b**) iNAP4 and **c**) SoNar ratios are not affected by 10 mM or 50 mM L-ascorbic acid or by 10 mM or 20 mM reduced glutathione in buffers of pH 7.2, 7.5, 8.1 or 8.4. $n = 3$; error bars ± maximum and minimal ratios.

a

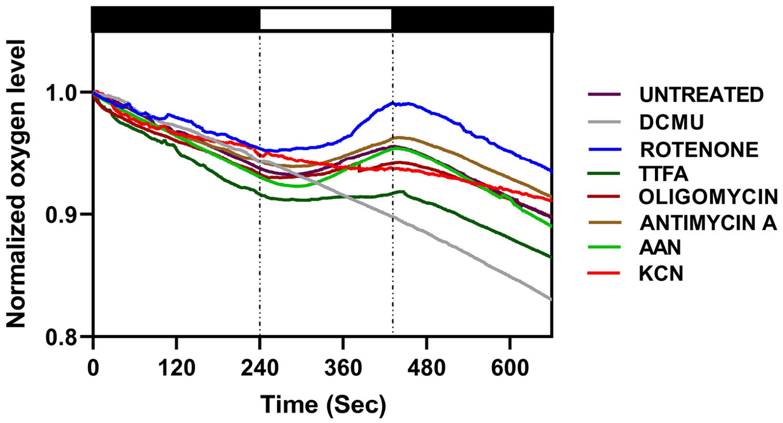

b

Inhibitors	Respiration rate (%)	Photosynthesis rate (%)
Untreated	100 ± 0	100 ± 0
DCMU	74 ± 3	3 ± 1
Rotenone	80 ± 9	74 ± 15
TTFA	78 ± 2	47 ± 4
Antimycin A	67 ± 10	78 ± 25
Oligomycin A	66 ± 6	47 ± 6
AAN	73 ± 7	63 ± 8
KCN	33 ± 3	6 ± 1

Supplementary Figure 10. **Oxygen levels of 10-d-old WT seedlings treated with various inhibitors. a**) Dynamic changes of normalized oxygen levels in seedlings treated with 0.02 mM DCMU, 0.05 mM rotenone, 0.1 mM TTFA, 0.01 mM oligomycin A, 0.01 mM antimycin A, 18 mM AAN or 0.5 mM KCN. Following an initial dark equilibration of the seedlings, oxygen was measured in the dark for 4 min before the seedlings were illuminated at an intensity of 296 µmol m^{-2} s^{-1} for 3 min at room temperature; this was followed by 3 min of darkness. The values under the black bars represented data obtained after light withdrawal at 180 s. **b**) Oxygen consumption rates (respiration) were calculated for the timeframe 480 s to 520 s while oxygen evolution rates were calculated for the timeframe of 300 s to 360 s. Photosynthesis rate was calculated as the oxygen evolution rate minus the oxygen consumption rate. The respiration rate (6.47 nmol O_2/min) and photosynthesis rate of WT control (12.02 nmol O_2/min) were set as 100% (n = 3; values are ± SEM).

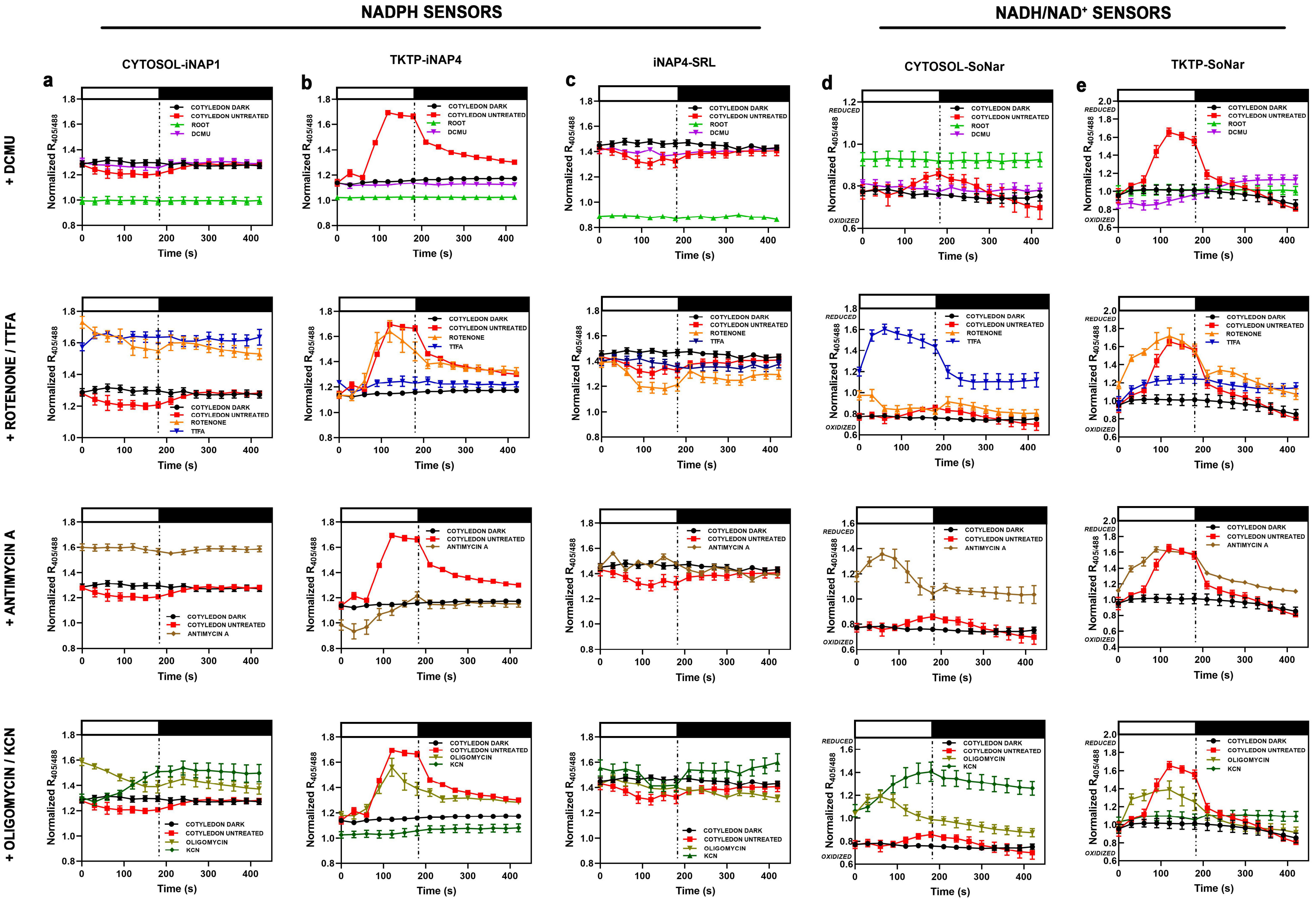
NADPH SENSORS
NADH/NAD⁺ SENSORS
CYTOSOL-iNAP1
TKTP-iNAP4
iNAP4-SRL
CYTOSOL-SoNar
TKTP-SoNar
a
b
c
d
e
+ DCMU
+ ROTENONE / TTFA
+ ANTIMYCIN A
+ OLIGOMYCIN / KCN
Normalized R405/488
Time (s)
REDUCED
OXIDIZED
COTYLEDON DARK
COTYLEDON UNTREATED
ROOT
DCMU
ROTENONE
TTFA
ANTIMYCIN A
OLIGOMYCIN
KCN

Supplementary Figure 11. Dynamic changes of NADPH levels and NADH/NAD$^+$ ratios in 10-d-old seedlings pre-treated with various inhibitors (0.02 mM DCMU; 0.05 mM rotenone; 0.1 mM TTFA; 0.01 mM AA; 0.01 mM oligomycin or 0.5 mM KCN). The normalized ratiometric changes of **a**) cytosol-iNAP1, **b**) TKTP-iNAP4, **c**) iNAP4-SRL, **d**) cytosol-SoNar and **e**) TKTP-SoNar ratios in cotyledon and root of 10-d-old seedlings in response to illumination up to 180 s at 296 μmol m^{-2} s^{-1} are presented under white bars. The values under the black bars represent data obtained after light withdrawal at 180 s (n = 5; error bar ± SEM). All results were normalized with the corresponding iNAPc subcellular sensors.

Supplementary Table 1. **List of PCR primers for vector construction.**

Primer	Sequence (5'-3')	Purpose
iNAP/SoNar-Forward	TAATGGATCCATGAACCGGAAGTGGGGCCT	Amplify iNAP/SoNar cDNA
iNAP/SoNar-Reverse	ATTATCTAGATTAGCCCATCATCTCCTCCCGCC	
iNAP/SoNar-SRL-Reverse	ATTATCTAGATCA**AAGACGACT**GCCCATCATCTCCTCCCGCCAC	Addition of peroxisomal targeting peptide (bold) to iNAP/SoNar cDNA
TKTPsp-Forward	CATTCATATGATGGCGTCTTCTTCTTCTCT	Amplify the chloroplastic-like targeting peptide of *Nicotiana tabacum* transketolase
TKTPsp-Reverse	ATTACTGCAGCGCAGTCTAGTTTTCTCTAT	
MT-Forward	ATTACATATGATGGCTTCTCGGAGGCTTCTC	Amplify the mitochondrial-targeting sequence from *Nicotiana plumbaginifolia* β-ATPase
MT-Reverse	TAATGAATTCACCAGCGCCGGTGAACTCAT	

Nucleotides complementary to the template DNA are underlined.
